# Bioturbation Affects Bioaccumulation: PFAS Uptake from Sediments by a Rooting Macrophyte and a Benthic Invertebrate

**DOI:** 10.1021/acs.est.4c03868

**Published:** 2024-11-11

**Authors:** Ioanna S. Gkika, Michiel H. S. Kraak, Cornelis A. M. van Gestel, Thomas L. ter Laak, Annemarie P. van Wezel, Robert Hardy, Mohammad Sadia, J. Arie Vonk

**Affiliations:** † Department of Freshwater and Marine Ecology (FAME), Institute for Biodiversity and Ecosystem Dynamics (IBED), University of Amsterdam, Science Park 904, 1098 XH Amsterdam, The Netherlands; ‡ Amsterdam Institute for Life and Environment (A-LIFE), Faculty of Science, Vrije Universiteit Amsterdam, De Boelelaan 1108, 1081 HZ Amsterdam, The Netherlands; § KWR Water Research Institute, P.O. Box 1072, 3430 BB Nieuwegein, The Netherlands

**Keywords:** environmental occurrence, environmental fate, plant uptake, macrophyte, oligochaetes, bioaccumulation factors, field-contaminated sediment

## Abstract

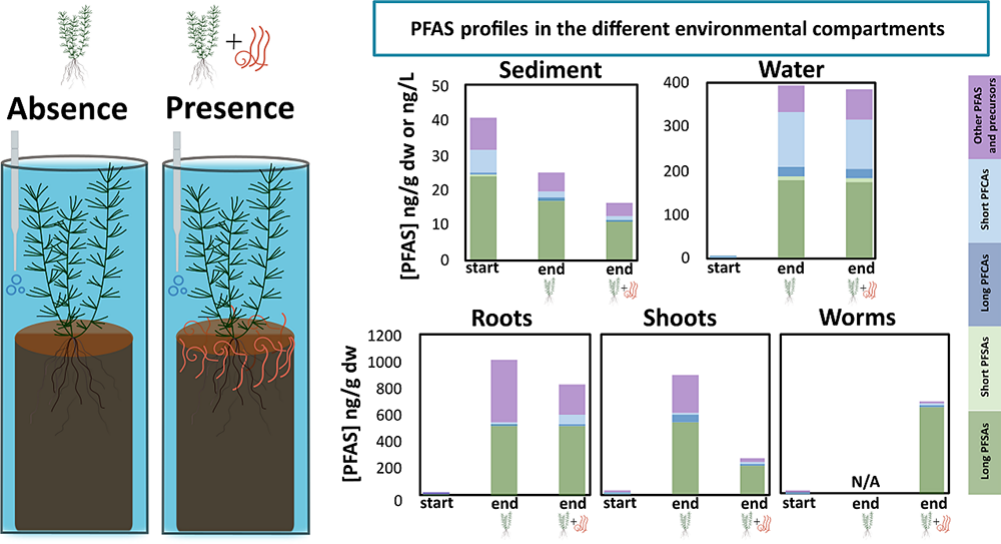

Despite the widespread presence of per- and polyfluoroalkyl substances (PFAS) in freshwater environments, only a few studies have addressed their bioaccumulation in macrophytes and benthic invertebrates. This study therefore aimed at investigating the presence of 40 PFAS in sediments, assessing their bioaccumulation in a rooting macrophyte (*Myriophyllum spicatum*) and a benthic invertebrate (*Lumbriculus variegatus*) and examining the effects of the presence and bioturbation activity of the invertebrate on PFAS bioaccumulation in the plants. The macrophytes were exposed to sediments originating from a reference and a PFAS-contaminated site. The worms were introduced in half of the replicates, and at the end of the experiment, PFAS were quantified in all environmental compartments. Numerous targeted PFAS were detected in both sediments and taken up by both organisms, with summed PFAS concentrations in organisms largely exceeding concentrations in the original sediments. Bioaccumulation differed between organisms and the two sediments. The presence of the worms significantly reduced the PFAS concentrations in the plant tissues, but for some compounds, root bioaccumulation increased in the presence of the worms. This effect was most prominent for the degradable PFAS precursors. It is concluded that organisms affect the environmental fate of PFAS, emphasizing that contaminant–macroinvertebrate interactions are two-sided.

## Abstract 2

This study reports the ubiquitous presence of various PFAS, their bioaccumulation by a macrophyte and a benthic invertebrate, and the influence of this invertebrate on their bioaccumulation, with a view to enhancing our understanding of PFAS behavior and eventually improving their environmental risk assessment.

## 1 Introduction

Per- and polyfluoroalkyl substances (PFAS) are man-made chemicals, designed to be water-, oil-, and stain-repellent, as well as persistent.^1^ These properties make PFAS suitable for a wide range of industrial applications, like surfactants, coatings, aqueous film-forming foams, as well as consumer products.^1^ Their persistency, however, is a chemical feature of environmental concern, since it can lead to prolonged exposure, bioaccumulation, trophic transfer, and consequent adverse effects on human and environmental health.^2,3^

Despite these worrisome characteristics, PFAS continue to be released into the environment on a large scale and have been detected in numerous abiotic^4,5^ and biotic^6,7^ matrices at considerable concentrations. A substantial number of studies have documented the occurrence of PFAS in sediments.^8,9^ However, the uptake of these compounds by aquatic plants from sediment remains understudied. The great majority of mechanistic mesocosm studies have focused on agricultural plants^10−16^ and soil-plant systems,^17,18^ since these have a higher relevance for human health. Based on these studies, root uptake is considered to be the primary pathway for PFAS to enter plants.^15,19^ PFAS can then reach the root vascular cylinder and from there migrate upward to the shoots.^20,21^ The few studies that have investigated PFAS uptake by rooting macrophytes from sediments have reported a high bioaccumulation potential of PFAS in plants, as well as different bioaccumulation patterns between roots and shoots, which were largely affected by the intrinsic properties of the compounds.^22−25^ It was also suggested that plant uptake favors linear PFAS isomers over branched ones.^26−28^ To determine how molecular properties affect PFAS uptake in rooting macrophytes, further research into the interactions between PFAS-contaminated sediments and aquatic plants is required, especially for a broader spectrum of PFAS than those studied so far.^29^

Sediment-bound PFAS can also be an important source of contamination for benthic invertebrates,^30−32^ which are at the basis of the aquatic food web. However, there are still only a few mechanistic studies available on the bioaccumulation of PFAS from sediments into aquatic or benthic invertebrates.^31−35^ Some studies have indicated the ability of sediment dwellers to affect the bioavailability of compounds through bioturbation.^36−39^ For PFAS, however, such research remains limited and to the best of our knowledge only one study has been performed, which suggested that the aquatic invertebrates *Hyalella azteca* and *Limnodrilus hoffmeisteri* enhanced the release of PFOS from the sediment.^40^ In addition to the bioturbation activity of benthic invertebrates, sediment properties such as organic carbon content may also affect the sorption capacity and consequently the environmental distribution of PFAS.^41^ Recent studies on PFAS adsorption to soil and bioaccumulation by plants suggest that these processes are concentration-dependent.^24,42^ This concentration-dependent sorption may affect PFAS bioavailability in sediment–water systems and thereby the distribution of compounds between sediment, water, and organisms. The limited information available on the bioaccumulation of PFAS by rooting macrophytes and benthic invertebrates from contaminated sediments and on how bioturbation can affect PFAS uptake kinetics and bioavailability emphasizes the importance of studying these processes for the wide variety of PFAS present in the environment.^29^

The aim of this study was, therefore, to investigate the effects of the presence of benthic invertebrates and their bioturbation activity on the bioaccumulation of various PFAS from field-collected sediments by a rooting macrophyte and the invertebrates themselves. To this end, we investigated (i) the presence of different PFAS classes in sediments collected from both reference and contaminated sites; (ii) the bioaccumulation of these PFAS by a rooting macrophyte (*Myriophyllum spicatum*) and a benthic invertebrate (*Lumbriculus variegatus*); and (iii) the impact of the presence of and bioturbation by the benthic invertebrate on PFAS bioaccumulation and redistribution in a sediment–plant system. Rooting macrophytes were exposed to sediments collected from the sites of interest under laboratory conditions. After 28 days of exposure, sediment-inhabiting worms were introduced into half of the replicates, and the experiment continued for another 28 days. PFAS concentrations were quantified in all compartments, including sediment, water, roots, shoots (in the presence and absence of worms), and worms. To the best of our knowledge, this is the first study to assess the effects of bioturbation on PFAS bioaccumulation and redistribution in a sediment–water system using a combination of a rooting macrophyte and a benthic invertebrate.

## 2 Materials and Methods

### 2.1 Sediment Sampling and Characterization

Sediment cores were collected from two lowland lakes (Figure S1). Details on the exact locations and sampling methods are described in Section S1 (Text S1). A total of eight cores per location were used for setting up the mesocosms, and another four cores per location were used for the sediment characterization (Text S2, Tables S1, and S2) and the determination of the initial PFAS load of the sediment. The sediment analyzed initially, before the start of the experiment, did not come into contact with water other than the water that was sampled together with it in the original sediment core taken from the field. This original overlaid water was then removed once the sediments were transferred to shorter cores (Text S1), which were then directly analyzed for PFAS.

### 2.2 Test Organisms and Experimental Setup

The rooting submerged plant *Myriophyllum spicatum* (Eurasian watermilfoil or spiked watermilfoil) grows in shallow, stagnant, or slow-moving water.^43^ Its wide geographical distribution and high ecological relevance for freshwater ecosystems have led to its broad use in environmental research.^43^ The specimens used in this experiment were cultured at the University of Amsterdam in aerated aquaria with artificial sediment (Text S3) and Dutch Standard Water (DSW; Text S4).

The oligochaete *Lumbriculus variegatus* (blackworm) is recommended by the U.S. EPA (2000)^44^ for assessing bioaccumulation of contaminants from freshwater sediments. *L. variegatus* lives in shallow marshes, ponds, and swamps, where it feeds on microorganisms and organic material. Worms were supplied by Romberg Aquariumhuis (NL) in 90 mL plastic bags filled with water. Prior to the experiment, worms were cultured in aerated aquaria containing artificial sediment and DSW.

To establish baseline PFAS concentrations, in addition to the four sediment cores per sampling site, four replicates of water, whole plants, and worms were collected separately, before the start of the experiment and frozen for subsequent PFAS analysis. At the start of the experiment (2nd March 2022), two *M. spicatum* shoots (∼7 cm length) were left to root in each of the eight sediment cores per lake, resulting in a total of 16 test cores. Each core (6 cm Ø) was immersed into a 4 L high-density polyethylene (HDPE) bottle that was filled with 3.5 L of DSW until the plants were covered (Figure S2). The bottles were placed in a climate room (20 °C, light:dark 16:8 h) and continuous aeration using a glass Pasteur pipet was installed in each bottle.

During the first 28 days of the experiment, the plants were allowed to grow, after which the bioturbating worms *L. variegatus* were introduced into half of the replicates from each location, and the experiment was run for another 28 days (Figure S2). It should be noted that the four replicates per site and per condition were not technical replicates, but rather field replicates, since each core was individually sampled in the field and brought into the laboratory intact, transferring local variability in sediment composition.

To obtain enough biomass for chemical analysis, a higher worm density than recommended by the OECD guidelines for bioaccumulation testing with sediment or soil inhabiting worms^45,46^ was chosen. To this end, 2.7 and 1.7 g (wet weight) of *L. variegatus* were added to the reference and contaminated sediments, respectively, corresponding to a density of approximately one worm per gram of dry sediment.

At the end of the 56 day experiment, from each individual core, the plants were harvested from the sediment and gently rinsed with Mili-Q water to remove sediment particles. The shoots were separated from the roots using scissors that were previously cleaned with methanol, and the dry weights after freeze-drying of both plant parts were recorded. Worms were harvested using a plastic Pasteur pipet and left to depurate for 48 h on wet filter paper in a Petri dish. This method of gut depuration has been described previously and is commonly used for oligochaetes.^45,47,48^ Sediment and water were subsampled, and all samples were stored at −20 °C until PFAS extraction.

### 2.3 PFAS Extraction and Analysis

All matrices were analyzed for 42 PFAS covering a wide range of structures, including six isomer pairs (Table S3). Individual branched isomers were not differentiated in the analysis, and the term “branched isomers” here refers therefore to the sum of all detected branched isomers per compound. From each of the four replicate cores, one water, sediment, root, shoot, and worm sample was analyzed per location for the initial PFAS concentrations at the start and at the end of the experiment for both the plant-only and plant-with-worm treatments. However, for the contaminated sediment exposures, worms from only one replicate could be extracted due to sample loss. PFAS concentrations in sediment and biota were expressed per dry weight.

The protocols used for PFAS extraction, analysis, and quality control assessment have been described previously.^49,50^ Briefly, for the water samples, a weak anion exchange solid phase extraction was applied. For the sediment and biota samples, solid–liquid extraction, followed by a weak anion exchange solid phase extraction and a cleanup step were performed. A detailed description of the extraction protocols for all matrices, the quantification method, and the quality assurance/quality control criteria can be found in Section S3 (Texts S5, S6, and Tables S4–S6). It should be noted that sediment concentrations here reflect total concentrations, consisting of both solid-sorbed and porewater-dissolved PFAS.

### 2.4 Bioaccumulation

The biota to sediment accumulation factor (BSAF) was used to describe the enrichment of PFAS concentrations in biota from the sediment, while the bioconcentration factor (BCF) was used to describe the ratio of PFAS concentrations between biota and water. All BSAFs and BCFs were calculated separately for each location and for all replicates (*n* = 4, unless stated otherwise). The BSAFs were calculated from eq 1 for the macrophyte roots and the worms, and the BCFs for the shoots were calculated from eq 2. In these equations [PFAS]_root_, [PFAS]_worm_, and [PFAS]_shoot_ correspond to the PFAS concentrations in each of these respective compartments at the end of the experiment and [PFAS]_sediment_^*t*=56^ and [PFAS]_water_^*t*=56^ correspond to the PFAS concentrations in the sediment and water at the end of the experiment.
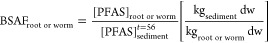
1
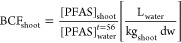
2

## 3 Results

### 3.1 Sediment Characterization

The two sediments had comparable nitrogen contents (Table S1) but differed in the organic carbon content and the concentration of organically bound phosphorus, with the latter being more than six times higher in the contaminated sediment compared to the reference sediment (9.39 and 1.48 mg/kg respectively). Grain size analysis revealed that the reference sediment mainly consisted of particles of size classes 125–250 μm and 250–500 μm, with almost equal parts, adding up to ∼87%, while the contaminated sediment was finer, containing more than 63% of size class 125–250 μm particles, followed by 32% of size class 63–125 μm particles (Table S2).

### 3.2 Occurrence of PFAS in Environmental Compartments and Organisms

**Figure 1:**
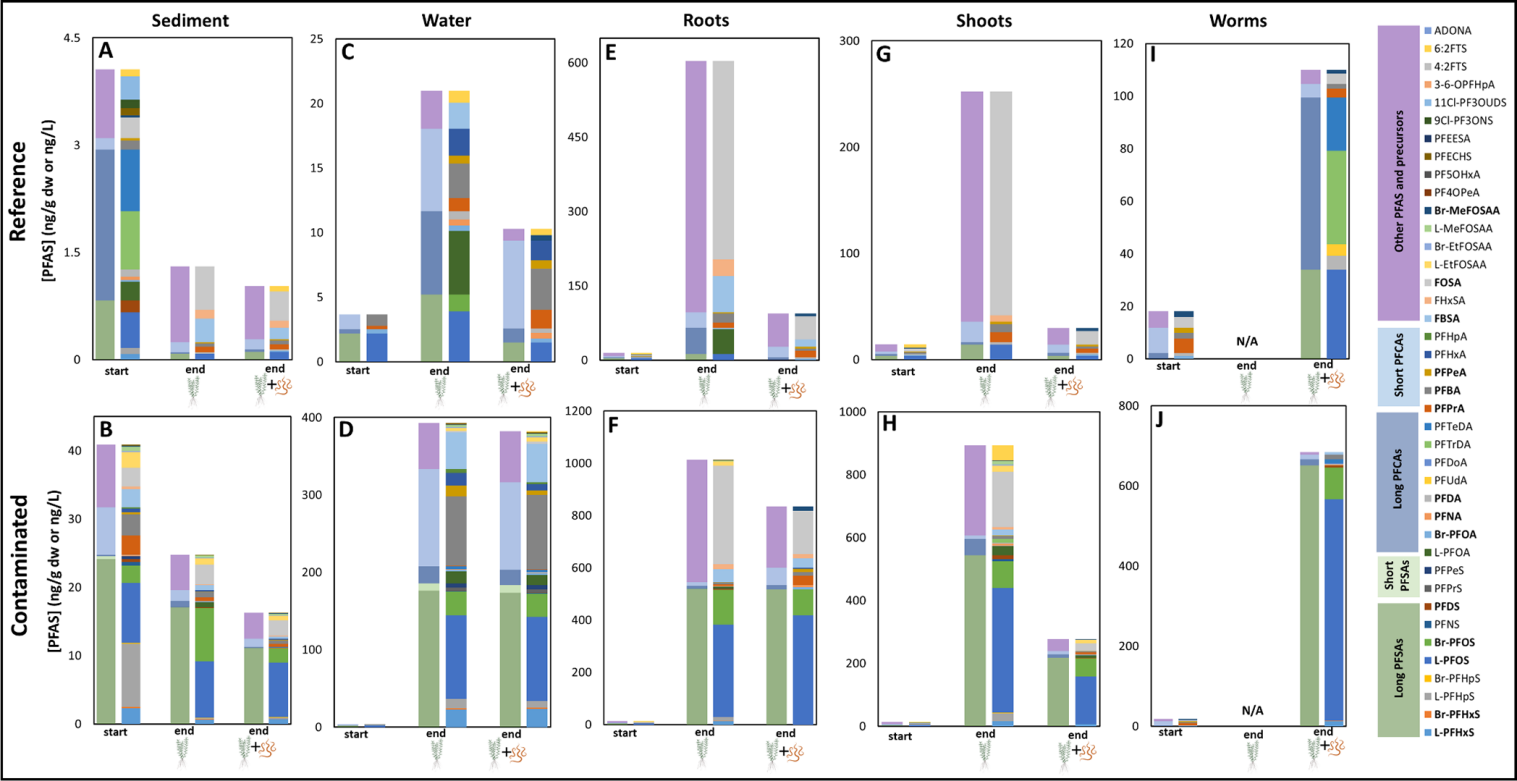
PFAS profiles and average (*n* = 4) individual PFAS concentrations in the different compartments (sediment, water, roots, shoots, and worms) at the start and the end of the 56 days mesocosm experiment in which plants (*Myriophyllum spicatum*) and worms (*Lumbriculus variegatus*) were exposed to reference (Gaasperplas) and contaminated (Blokkersdijk) sediments. For visualization purposes, error bars are not plotted in the graphs, but the raw data behind the bar plots are included in Tables S7–S9. In each pair of bars, the left one shows the different PFAS subclasses, while the right one depicts the individual compounds that belong to each subclass. The different treatments (absence or presence of worms) are indicated below the stacked bars with the respective icons. In the legend, the compounds that were detected in all compartments (sediment, water, plant, and worms) in at least one of the locations and for at least one time point are highlighted in bold. Results for worms shown in plot *J* are based on only 1 replicate. Please note that the scales of the *y* axes differ between the panels, to enable visualization.

Data for 40 out of 42 target PFAS (Text S6) are reported for the collected sediments from the two locations, the used overlying water source, and both test organisms. All matrices contained PFAS both at the start and the end of the experiment, with variable shifts in the PFAS profiles and the individual PFAS concentrations in all matrices (Figure 1).

#### 3.2.1 PFAS Concentrations in Sediment and Water

In the reference field collected sediment, PFAS profiles were dominated by long-chain perfluorocarboxylic acids (PFCAs), followed by precursors and long-chain perfluorosulfonic acids (PFSAs) (Table S7 and Figure 1A). A total of 18 compounds were detected with a ∑PFAS concentration of 4.06 ng/g dw, and individual concentrations ranging from 0.023 (Br-PFOA) to 0.86 ng/g dw (PFTeDA). At the end of the 56 day experiment in the presence of the macrophytes, the ∑PFAS decreased to 32% (1.31 ng/g dw) of the initial concentration. The number of detectable PFAS decreased to eight, with PFAS profiles now being dominated by precursors (Table S8 and Figure 1A). The presence of both macrophytes and benthic invertebrates resulted in an even lower ∑PFAS concentration (1.03 ng/g dw; 25% of the initial concentration), also dominated by precursors (Table S9 and Figure 1A).

The contaminated sediment contained 10 times more PFAS than the reference sediment (∑PFAS = 40.9 ng/g dw). More substances were detected (24 versus 18) in higher individual concentrations (up to 9.23 ng/g dw for L-PFHpS) and with a different composition, since the profile was dominated by long-chain PFSAs, followed by precursors and short-chain PFCAs (Table S7 and Figure 1B). The ∑PFAS concentrations decreased to 60% of the initial concentration (∑PFAS 24.8 ng/g of dw) in the presence of the plants and to 40% of the initial concentration (16.3 ng/g of dw) when both organisms were present (Tables S8 and S9). The PFAS profiles remained similar in the contaminated sediment (Figure 1B), in contrast to those in the reference sediment.

At the start of the experiment, four PFAS (∑PFAS concentration 3.66 ng/L) were detected in the DSW that was added on top of the sediments (Table S7 and Figure 1C). Upon contact with the sediment from the reference location in the presence of the plants, the number of compounds increased to 12, and the ∑PFAS was more than six times higher (21.0 ng/L) than at the start of the experiment (3.66 ng/L), while the profile became more varied, including short- and long-chain PFCAs, followed by long-chain PFSAs. The joint presence of macrophytes and worms resulted in a lower increase in ∑PFAS (10.3 ng/L) in the overlying water, with a comparable number of compounds (10) and a slightly changed profile, dominated by short-chain PFCAs and with less precursors and PFSAs compared to the macrophyte-only treatment (Tables S8, S9, and Figure 1C).

In the replicates containing the PFAS-contaminated sediment, the ∑PFAS concentration in the water increased more than 100 times after 56 days, both in the presence of plants and in the presence of both organisms (392 and 382 ng/L, respectively). Over this period, the number of compounds in the water increased from four to 25 and 27, respectively (Tables S8, S9, and Figure 1D). The PFAS profiles were similar in both treatments at the end of the experiment, consisting mostly of long-chain PFSAs and short-chain PFCAs.

#### 3.2.2 PFAS Concentrations in Macrophytes and Worms

At the end of the exposure, macrophytes were in good condition, showing no signs of necrosis. The average dry weight of the roots was 0.083 and 0.12 g and that of the shoots was 2.0 and 3.5 g for the reference and contaminated sediments, respectively. Worms were in good condition, and comparable amounts were recovered from both types of sediments. In the original *M. spicatum* plants, eight out of the 40 PFAS were detected (Table S7 and Figure 1E,F), with a low ∑PFAS concentration (14.5 ng/g dw). Roots grown in the reference sediment in the absence of the worms contained a 41-times higher ∑PFAS concentration (603 ng/g dw) than that of the original plant tissue. The profile consisted of 10 detectable PFAS, mainly precursors and differed from the PFAS composition of the original sediment. The PFAS concentrations in the roots that were exposed to the reference sediment in the presence of worms had similar profiles as in the plant-only treatment, but the ∑PFAS was more than six times lower (94.2 ng/g dw) (Tables S8, S9, and Figure 1E). In the shoots, the ∑PFAS concentration after 56 days was over two times lower than in the roots (252 ng/g dw), but with similar profiles and number of detected compounds (eight) (Table S8 and Figure 1G). In the presence of the worms, the ∑PFAS concentration in the shoots was almost nine times lower (29.5 ng/g dw; eight compounds) compared to the plant-only treatment and was three times lower than in the roots, nonetheless with similar profiles, dominated by precursors (Table S9 and Figure 1G).

Roots exposed to the contaminated sediment in the absence of the worms accumulated almost exclusively long-chain PFSAs and precursors, consisting of 23 compounds, more than twice as many as in the roots of the plants grown on the reference sediment (10) (Table S8 and Figure 1F). The ∑PFAS concentration was 1.01 μg/g dw, 72 times higher compared to the initial concentration in the plants, and almost twice as high as in the roots grown on the reference sediment. In the presence of both organisms, PFAS profiles were similar, but the number of detected PFAS decreased to almost half (12) of the number in the plant-only treatment (23), while the ∑PFAS concentration was only slightly lower (834 ng/g dw) (Table S9 and Figure 1F).

In the shoots of the plants exposed to the contaminated sediment in the absence of worms, the PFAS profiles and the number of detected compounds (22) were similar to those in the roots (Table S8 and Figure 1H). The ∑PFAS (893 ng/g dw) was 61 times higher than in the original plants but lower than in the roots. When the worms were also present, fewer compounds were detected (14), the ∑PFAS was three times lower (278 ng/g dw) and the profile changed, consisting almost exclusively of long-chain PFSAs (Table S9 and Figure 1H).

Prior to contact with the sediments, the worms contained seven PFAS, mostly short-chain PFCAs, with a ∑PFAS concentration of 18.2 ng/g dw (Table S7 and Figure 1I,J). After exposure to the reference sediment, a similar number of compounds was detected in the worms (nine), but the ∑PFAS concentration increased more than six times (110 ng/g dw), while the PFAS profile was now dominated by long-chain PFCAs and PFSAs (Table S9 and Figure 1I). After exposure to the contaminated sediment, 11 PFAS were quantified in the worms, almost exclusively long-chain PFSAs (Table S9 and Figure 1J), resulting in a ∑PFAS concentration of 684 ng/g dw, 38 times higher than at the start of the experiment and six times higher than in the worms exposed to the reference sediment.

#### 3.2.3 Mass Balance

To investigate how the compounds distributed between the sediments, plants, worms, and overlying water, mass balances were calculated. The details behind the calculations are presented in the SI (Section S5 and Text S7). Overall, recoveries of most PFAS were below 100%, except for L-PFOS, Br-PFOS, and FOSA (Table S10).

### 3.3 Bioaccumulation Factors

#### 3.3.1 PFAS Bioaccumulation Factors for Macrophytes and Worms

**Figure 2:**
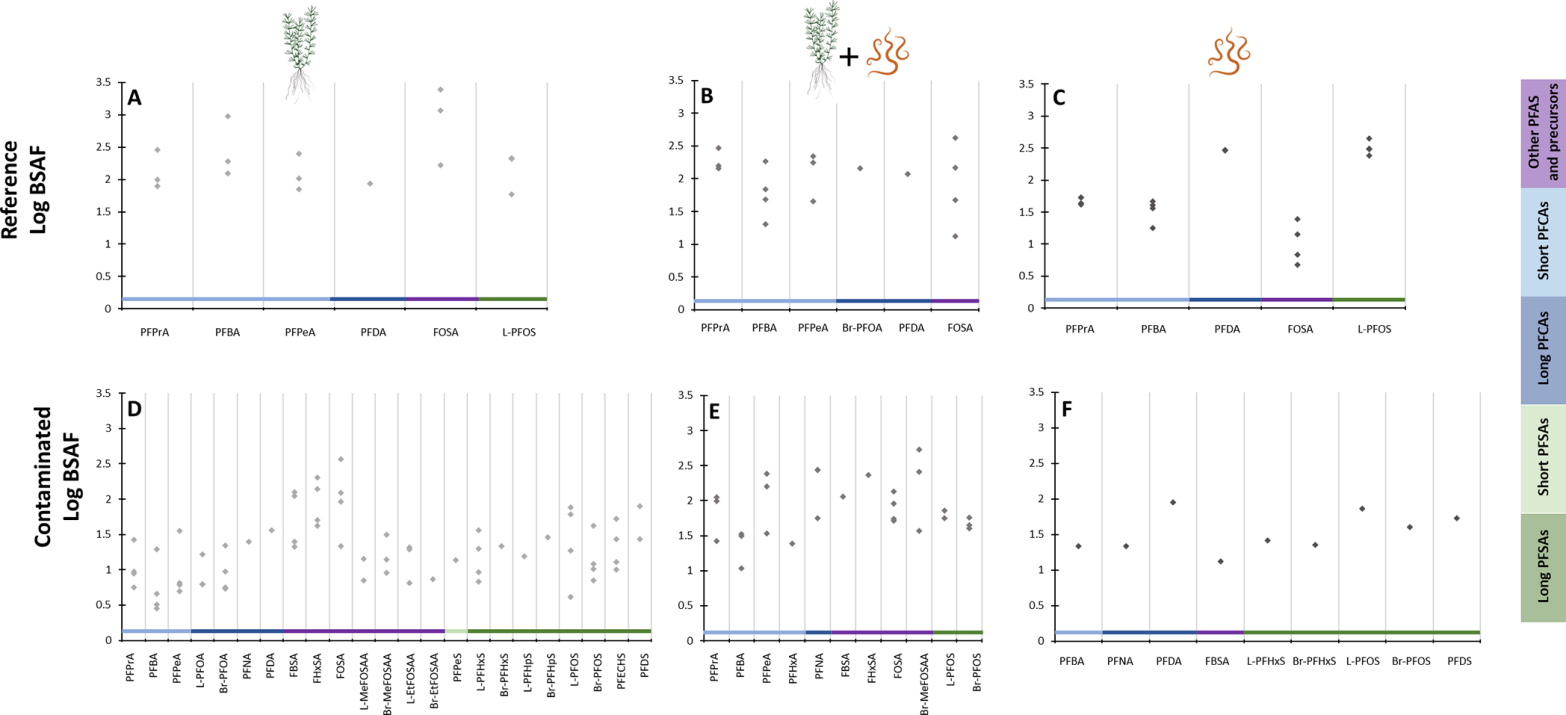
Logarithmically transformed biota to sediment bioaccumulation factors (BSAFs) for the uptake of PFAS from reference (Gaasperplas) and contaminated (Blokkersdijk) sediment into the roots of *Myriophyllum spicatum* [kg sediment dw/kg root dw] in the absence (A and D, respectively) and presence of worms (*Lumbriculus variegatus*) (B and E, respectively) and into the worms [kg sediment dw/kg worm dw] themselves (C and F, respectively). Each data point represents one replicate. The horizontal color bars indicate the different PFAS subclasses, with the compounds per subclass plotted with increasing number of fluorinated carbons. In general, *n* = 4, except for some compounds that were not detected in all replicates, as can be seen in Tables S11–S13. BSAFs in worms shown in plot *F* are based on only 1 replicate.

Logarithmically transformed biota to sediment accumulation factors were obtained for roots in the absence (BSAFs_root–_) or presence of worms (BSAFs_root**+**_) and for worms (BSAFs_worm_), all in kg sediment dw/kg root or worm dw (Tables S11–S13 and Figure 2). For the reference sediments, average BSAF values could be calculated for six PFAS both in the absence and in the presence of worms (Figures 2A,B). The compound that exhibited the highest average BSAF values was different between the two treatments with a higher value for the plant-only treatment (FOSA; 1,053) compared to the joint presence (PFPrA; 199). For worms, BSAFs could be calculated for five PFAS (Figure 2C). In the worms generally lower values were observed compared to the macrophyte roots, with the maximum average value reaching 326 (L-PFOS). For the contaminated sediments in the plant-only treatment, BSAFs could be calculated for 23 PFAS (Figure 2D), while in the joint presence of both organisms, they could be calculated for less than half of this number (11) (Figure 2E). In both cases, the highest average BSAF values were found for precursors (FOSA; 150 in plant-only treatment; FHxSA; 233 in worms and plant treatment). Similar to the reference sediment, BSAFs for worms generally were lower and PFDA showed the highest average BSAF of 91 (Figure 2F). Overall, for both sediment types, precursors tended to bioaccumulate more in the macrophyte roots and much less in the worms. The BSAFs for linear and branched isomers did differ in most cases, but these differences were not consistent.

#### 3.3.2 Comparison of Locations

Comparing the bioaccumulation behavior of individual PFAS, as well as PFAS subclasses between the two locations, revealed some differences. For plants grown in the reference sediment, BSAFs_root–_ for PFCAs was not correlated with chain length (Figure 2A), while for the contaminated sediment, BSAFs for PFCAs seemed to increase with the increasing chain length (Figure 2D). For the worms, no relationship between bioaccumulation and chain length was observed for the reference nor for the contaminated sediment (Figures 2C,F). For the organisms exposed to the highly contaminated sediment, the PFAS concentrations were higher than in the reference sediment, which enabled the quantification of BSAF values for more PFAS. However, the BSAFs were generally lower for the contaminated sediment compared to the reference sediment for both organisms. Comparing the difference in bioaccumulation between the two organisms was not possible as little overlap was found between the PFAS present in the two organisms and almost no overlap between the two locations (only one compound) (Figure S3).

### 3.4 Effect of Bioturbation on PFAS Bioaccumulation by Macrophytes

**Figure 3:**
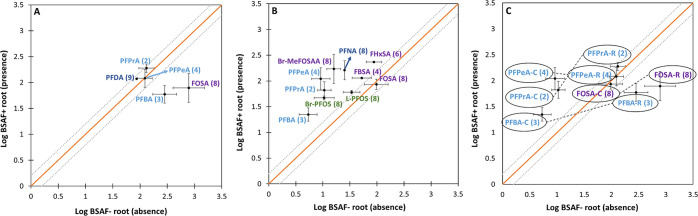
Effect of the presence of worms (*Lumbriculus variegatus*) on the bioaccumulation of PFAS in plant roots (*Myriophyllum spicatum*) following exposure to field sediments from two locations. Average (±SEM) LogBSAFs for PFAS bioaccumulation in roots in the presence and absence of worms are plotted for the reference (Gaasperplas) (A) and contaminated (Blokkersdijk) (B) sediments (*n* = 4, except with missing error bars *n* = 1). In panel C, BSAFs for PFAS overlapping between the two locations are plotted together, and the compounds of each pair are connected with each other with a dashed line. The names of the compounds corresponding to each data point are presented and the number in parentheses indicates the number of fluorinated carbons. The letters R (reference) and C (contaminated) in the last panel indicate the locations. All BSAF values are given in kg sediment dw/kg root dw. The solid orange line indicates the 1:1 line, and the dotted gray lines indicate the margin of ±0.2 log units, which was used to evaluate the distance of the compounds from the 1:1 line.

To evaluate the effect of the presence of the benthic invertebrate and its bioturbation activity on the bioaccumulation of PFAS by the macrophytes, the bioaccumulation in the roots in the presence (BSAF_root**+**_) and absence (BSAF_root–_) of the worms in the sediments from both locations (Figure 3) was compared. Only compounds that were concurrently present in the roots of both treatments (absence or presence of worms) and for which BSAFs could be calculated are plotted. In the scatterplots, two categories were distinguished based on the distance of the data points from the 1:1 line (*X* = *Y*) shown in orange. The first category included compounds that were on or close to the 1:1 line, indicating similar bioaccumulation factors between the two treatments. These data points were within a ±0.2 log unit range from the 1:1 line. The second category concerned compounds that deviated more than 0.2 log units from the 1:1 line. For the reference site (Figure 3A), we could compare the BSAFs for five compounds, two of which (PFBA and FOSA) deviated strongly from the 1:1 line and were positioned below it, indicating a higher bioaccumulation in the plants in the absence of worms (Figure 3A). The BSAFs in the macrophytes for the remaining three compounds, consisting of two short- and one long-chain PFCAs, were similar in the presence or absence of the worms. In the contaminated sediment, the effect of the worm presence could be evaluated for 10 PFAS (Figure 3B), showing patterns that were opposite to those observed for the reference sediment. Three out of these 10 PFAS (two precursors and one long-chain PFSA) were positioned on or close to the 1:1 line, indicating comparable bioaccumulation between the two treatments. The remaining seven compounds were positioned above the 1:1 line, suggesting higher bioaccumulation in the presence of worms. Finally, there were only four compounds for which BSAFs could be compared in the presence and absence of worms for both the reference and contaminated sediment (Figure 3C), all of which exerted opposite behavior between the two types of sediments. More specifically, compounds for which the BSAFs of plants growing in the contaminated sediment did not seem to be affected by the presence of the worms were impacted in the reference sediments and *vice versa*. On top of that, BSAFs from the contaminated sediments that were influenced by the presence of worms exhibited higher values in the presence of worms, while the opposite was observed for bioaccumulation into the plants from the reference sediment. Finally, the shoot bioconcentration factors from water, expressed as BCF, in the absence and presence of worms are presented in the SI (Text S8 and Figure S4).

## 4 Discussion

This study presented the occurrence and profiles of PFAS in reference and contaminated freshwater sediments and their bioaccumulation by rooting macrophytes and a benthic invertebrate. The experimental design allowed for assessment of the effect of the presence and bioturbation activity of the benthic invertebrate on PFAS bioaccumulation and redistribution in the sediment–water–macrophyte system.

### 4.1 Presence of PFAS from Different Subclasses in Freshwater Sediments

The need to broaden the spectrum of targeted PFAS has been underlined by earlier research.^29,51−53^ This study therefore examined PFAS structures which are more rarely screened for in PFAS monitoring and experimental studies, including ultrashort PFAS, branched isomers, chlorinated polyfluoroether sulfonic acids, as well as a cyclic sulfonic acid. Despite this extensive PFAS list, we acknowledge that the actual number of potentially present PFAS remains higher, being either unknown precursors or simply unscreened structures. The presence and potential (bio)transformation of unknown precursors could also have contributed to the increased concentrations of PFAAs observed at the end of the experiment. In the reference sediments, more than 40% of the targeted compounds were quantified, underlining that there may be hardly any reference site concerning PFAS. Comparing the presently measured PFAS concentrations in the reference sediments with those from earlier studies^8^ revealed similar levels, although slightly different PFAS were screened for. The PFAS concentrations in contaminated sediments reported in the literature vary greatly, which is to be expected since different PFAS sources result in distinct PFAS fingerprints.^54^ Nevertheless, qualitative comparisons showed that in one case, PFAS concentrations were similar to those currently measured,^55^ but in other sediments, the concentrations reported were orders of magnitude higher compared to those detected in the present study.^56−58^ It is concluded that broadening the spectrum of targeted PFAS indeed revealed the presence of many of these, which may also imply that many more unmeasured PFAS could be present in the environment, where profiles and concentrations are location- and source-specific. Acknowledging the fact that screening for numerous PFAS in various environmental matrices is expensive and labor intensive, a monitoring list that includes at least some representatives from the different subclasses is proposed.

### 4.2 Bioaccumulation of PFAS by a Rooting Macrophyte and a Benthic Invertebrate

The findings of the present study suggest that many PFAS do have high bioaccumulation potential. From the 18 PFAS, for which BCFs for shoots were calculated for both sampling sites in the absence of worms, eight had values >5000 L/kg in at least one or both locations, reaching up to 97,695 L/kg shoot dw for FOSA. Based on the PBT/vPvB assessment criteria set by ECHA (2023),^59^ these compounds, which in our case included mostly PFSAs and precursors, are categorized as “very bioaccumulative” and therefore require stricter regulations compared to compounds not categorized as PBT/vPvB. Although a comparison for the BSAF values was not possible, due to their absence in the available literature, based on the calculated values, reaching up to 1,053 kg sediment dw/kg root dw for FOSA, it may be argued that at least some PFAS subclasses have a high bioaccumulation potential from sediments to macrophytes.

Although PFAS uptake by terrestrial plants has previously been reported,^60^ information about PFAS bioaccumulation in aquatic macrophytes remains limited.^29,61,62^ In a recent review gathering BSAFs for PFAS, almost no data on macrophytes were reported,^63^ since the few studies that did investigate PFAS uptake by rooting macrophytes from sediments reported only BCFs from water.^21,23−25^ Concerning the benthic invertebrates, there are some studies on bioaccumulation from field-collected and PFAS-spiked sediments.^31−35,64^ We compared our BSAF data for *L. variegatus* with previously reported BSAFs for the same organism after harmonizing all units (Table S15). Our data were on the lower end of the BSAF calculated in another study using field-contaminated sediment,^32^ while they were much lower compared to the other two studies.^33,64^ A major distinction that could have led to these discrepancies is the presence of the macrophytes, which may have competed with the worms for PFAS uptake. Moreover, one of the studies was performed with spiked, artificial sediment,^64^ where bioavailability of organic compounds is expected to be higher compared to field-contaminated sediments that have been in contact with the contaminants for an extensive time period.^65,66^ It should be realized that BSAFs can only be reliably calculated when the system is in steady state, which was only addressed by Higgins et al.,^33^ observing a lack of equilibrium for several PFAS after 28 days. Our experimental setup did, however, not allow us to evaluate whether equilibrium was reached. The water content of the worms could be another confounding factor, specifically for the BSAF calculation of the short-chain compounds, which might have contributed to the high BSAF observed for PFPrA. On top of that, because the worms were not rinsed prior to extraction, any PFAS present in the water attached to the worms, as well as the internal water content of the worms, may have contributed to the body burden of the worms. Yet, assuming the steady state between overlaying water, worms, and pore water, this contribution was estimated to be low to negligible. However, this steady-state assumption cannot be verified, and therefore, BSAF comparisons even between studies on the same species, like that performed here (Table S15), should be interpreted with some caution. Additional differences in terms of the PFAS load and profiles, as well as the properties of the sediments could further explain the variation in the BSAFs. We chose not to compare BSAFs reported for other benthic invertebrates, since on top of the aforementioned factors, there is additional variation related to the taxonomy, physiology, feeding behavior, and exposure route of the organism based on its habitat, living either in or on top of the sediment.

The BSAF calculations were based on the final PFAS concentrations in the sediment measured at the end of the experiment. It should be noted, however, that these BSAFs may be correct only for compounds that reached steady-state, while they may have been overestimated for the ones that did not. Yet, our experiment did not allow for assessing PFAS desorption kinetics from the sediment or uptake kinetics in the plants and worms. Nonetheless, using the arithmetic mean of the sediment concentrations measured at the start and end of the experimental period still resulted in high values, highlighting the high bioaccumulation potential of PFAS (Table S14).

The bioaccumulation of PFAS in the roots and the worms in relation to their chain length was not consistent between the two locations nor for the two treatments in our study. In some cases, increased bioaccumulation in relation to the fluorinated chain length was observed for the carboxylic acids and some sulfonamide-based precursors (contaminated sediment roots in the absence of worms), which aligns with earlier research,^23,32^ but in other cases, this relationship was absent. Absence or even negative relationships between bioaccumulation and PFAS chain length have also been reported previously concerning bioaccumulation factors based on soil, instead of porewater concentrations.^15,21,67^ Two main explanatory factors were proposed for the different relationships between bioaccumulation and chain length that are often reported in the literature. Although these explanations originate from terrestrial studies, they could also apply to aquatic systems such as the one in our study. The first explanation concerns variations in root structures among plants. These different root structures could lead to distinct interactions with contaminants,^68^ as well as adsorption/absorption mechanisms of pollutants, which can involve, among others, root exudates, ion channels and/or aquaporins.^69−72^ The second one relates to the inability to differentiate between the strong external adsorption of long-chain and the absorption of shorter-chain PFAS.^11^ Based on these findings, PFAS uptake by plants seems to be compound- and species-specific. However, our setup did not allow for differentiation between external plant sorption or internal plant uptake; therefore, the second explanatory factor could partly justify the inconsistency of the observed bioaccumulation patterns between the different compounds. Relationships between bioaccumulation and chain length may be further obscured by differences between compounds in reaching steady state, as might have been the case in our study.

Similar PFAS profiles and trends were observed in roots and shoots, but we cannot unravel the processes resulting in these profiles and trends. Our setup did not allow us to distinguish PFAS that bioconcentrated from the water to the shoots and compounds that could have translocated between roots and shoots. Earlier research reported positive correlations between root and shoot concentrations^70,73^ with submerged species having higher BCFs than free floating ones.^23^ Merging these results with our observations suggests that it was the combination of both mechanisms (i.e., uptake from the water phase and translocation from the roots) that contributed to the exposure of the shoots in this study and the resulting PFAS bioaccumulation profiles. This implies that the calculated BCFs may have overestimated the PFAS bioaccumulation potential from water to the shoots (Table S13).

The differences in the bioaccumulation patterns observed between the two locations could, to some extent, be attributed to the different sediment characteristics and to concentration-dependent uptake. The organic carbon contents of the two sediments, the grain size distribution, and other characteristics were quite distinct (Tables S1 and S2), causing potential differences in PFAS bioavailability. The lower organic carbon content and the lower clay content of the reference sediment could result in a weaker adsorption of the PFAS to this sediment compared to the contaminated one, resulting in higher bioavailable concentrations. In addition, the aqueous chemistry (pH and ionic strength), composition, and concentrations of PFAS differ between the field-contaminated sediments, affecting the bioaccumulation. Moreover, the concentration of organically bound phosphorus (P_org_) was more than six times higher in the contaminated than in the reference sediments (Table S1). The higher nutrient availability increased plant growth on the contaminated sediment compared to the reference sediment, as indicated by the higher (dry) weights. The difference in the trophic state between the two locations may have affected the distribution and bioaccumulation of PFAS due to biomass dilution, as previously reported for polycyclic aromatic hydrocarbons (PAHs).^74^ All of these factors possibly also played a role in the way the presence of the worms affected the PFAS bioaccumulation. However, the limited overlap between the compounds taken up in the presence and absence of worms in both sediments hampers firm conclusions.

Another factor that could have played a role in the different patterns observed between the two sediments is the initial PFAS load. The contaminated sediment contained approximately 1 order of magnitude higher ∑PFAS concentrations and the subclass of long-chain PFSAs was much more dominant, compared to the reference sediment, where PFCAs were the most abundant, followed by PFSAs and precursors with almost equal contributions. Competing uptake mechanisms have previously been reported for PFAS bioaccumulation,^73,75,76^ while various studies suggested that PFAS uptake by plants is concentration-dependent.^24,42^ Concentration dependency of bioaccumulation factors has been proposed for metals,^77^ as well as for PFAS.^78^ In the latter study, where the bioconcentration factors from water to green mussels were assessed at various PFAS concentrations, lower BCFs were observed for the organisms exposed to higher PFAS concentrations. This inverse relationship was supported by the nonlinear adsorption mechanism underlining the uptake of the investigated compounds.^78^ Also, in this work, generally lower BCFs were observed for the organisms exposed to the highly contaminated sediment, which further supports the inverse relationship between the exposure concentration and bioaccumulation.

In the present study, the wide variety of PFAS exhibited different bioaccumulation degrees between different organisms, locations, and treatments. It is therefore challenging to assign a general bioaccumulation potential to each PFAS subclass. Nonetheless, this work showed that a wide variety of PFAS present in the environment appear to have a high bioaccumulation potential. However, the model describing the bioaccumulation potential of this diverse group of compounds likely contains many variables, including, on top of their molecular properties, factors such as sediment/soil properties, species interactions, total PFAS loads, as well as interactions with other chemicals.

### 4.3 Effect of the Presence and Bioturbation by the Benthic Invertebrate on PFAS Redistribution and Plant Bioaccumulation

Even though the assessment of the bioaccumulation in the worms from the contaminated sediment was based on only one replicate, we were still able to assess the impact of the presence of the worms on the distribution and plant uptake of PFAS. Our findings revealed that the presence and bioturbation activity of the benthic invertebrate did affect the distribution of PFAS over the system, and in the presence of the worms, the ∑PFAS concentration in the plant roots and shoots was lower. This was the case for both locations but was more prominent for the plants grown on the reference sediment, where the initial PFAS load of the sediment was much lower. This suggests that there was competition for PFAS uptake between the organisms, as proposed by other studies^73,75,76^ or that some compounds had higher affinity for the worms compared to the macrophyte. This is supported by the BSAF values for the plants at the reference sediment that for some compounds were lower in the presence of the worms. In our test system, worm densities were on the high end of the abundances found in natural environments. This may have had an effect on the relative distribution and plant uptake of PFAS. However, examining how different worm densities could affect PFAS environmental fate was beyond the scope of our study.

For the organisms exposed to the contaminated sediment, the PFAS load seemed to be so high that even in the presence of both organisms, the plants and especially the roots were still able to take up considerable amounts of PFAS. In the contaminated sediment, the concentrations of FOSA and L-PFOS remained relatively constant over the 56 day period, in both treatments, while all other compounds seemed to be depleted by the uptake by the organisms or by the release into the overlaying water. This could imply that the sediment had a high accessible stock of these two PFAS and once the readily bioavailable concentration was taken up by the organisms, it was constantly being replenished. This replenishment of depleted bioavailable concentrations via diffusion processes has previously been reported.^79,80^

The reduction in terms of the number of PFAS present in the plant roots in the presence of worms was more noticeable for the contaminated sediment. In the contaminated sediment, the root bioaccumulation of mainly PFCAs was affected by the presence of the worms, which could indicate that bioturbation made them more bioavailable to the macrophyte roots. This is supported by the BSAF values for the plants in the contaminated sediment that for some compounds were higher in the presence of the worms. The remaining non-precursor compounds that were not found in the roots when worms were present were found either in the worm tissues or in the overlaying water. The presence of the worms notably reduced the concentrations of precursors in the roots as well as shoots, while very few to no precursors were detected in the worms themselves at both locations. These observations could relate to the increased oxidation conditions caused by the bioturbation activity of the worms, which could have enhanced the (bio)transformation of some precursors into PFSAs. Earlier research has shown that invertebrates are capable of biotransforming PFAS precursors, including N-EtFOSAA and FOSA to PFSAs.^33,81^ As previously mentioned, unmonitored PFAS including precursors could also have contributed to the increased concentrations of “terminal” PFAS observed at the end of the experiment. This is also illustrated by the calculated mass balances, where the observed mass balances above 100% might be due to the potential (bio)transformation of precursors into linear and branched PFOS in our systems, especially in the presence of the worms. Our setup did not allow for differentiation between external transformation or biotransformation within the organisms. Yet, we were able to demonstrate that the presence of the worms and their bioturbation had an impact on the PFAS profile in the plants.

Another way in which bioturbation could have affected PFAS bioaccumulation is through alterations in the benthic microbial community and biogeochemistry.^82^ Microbiota are able to accumulate PFAS, but they could also play a role in PFAS degradation^70^ and redistribution from the sediment to the different environmental compartments.^22,83^ Contaminant-macroinvertebrate interactions, driven by the interplay between contaminant properties and macroinvertebrate traits, have been reported for other compound groups,^39^ and the present study showed that there are also various ways through which benthic invertebrates can interfere with the environmental redistribution and bioaccumulation of sediment-associated PFAS. It is therefore concluded that organisms affect the environmental fate of PFAS, highlighting that contaminant-macroinvertebrate interactions should be viewed as a bilateral relationship.

## Supporting Information Available

Sediment sampling and characterization, experimental setup, extraction and quantification of PFAS, PFAS concentrations in all matrices, mass balance analysis, PFAS bioaccumulation factors, bioaccumulation in plants versus animals, and effect of bioturbation on the bioconcentration from water to shoots (PDF)
