# Supplementary material for: Bioturbation Affects Bioaccumulation: PFAS Uptake from Sediments by a Rooting Macrophyte and a Benthic Invertebrate
Source: Environ Sci Technol. 2024 Nov 11;58(46):20607–18. doi: 10.1021/acs.est.4c03868 (PMC11580174; doi:10.1021/acs.est.4c03868)
Supplement: Supplementary file 1 — es4c03868_si_001.pdf [file es4c03868_si_001.pdf]

Supporting Information

Bioturbation affects bioaccumulation: PFAS uptake from sediments by a rooting macrophyte and a benthic invertebrate

Ioanna S. Gkika [a*], Michiel H.S. Kraak [a], Cornelis A.M. van Gestel [b], Thomas L. ter Laak [a, c], Annemarie P. van Wezel [a], Robert Hardy [a], Mohammad Sadia [a], J. Arie Vonk [a]

[a] Department of Freshwater and Marine Ecology (FAME), Institute for Biodiversity and Ecosystem Dynamics (IBED), University of Amsterdam, Science Park 904, 1098 XH Amsterdam, the Netherlands

[b] Amsterdam Institute for Life and Environment (A-LIFE), Faculty of Science, Vrije Universiteit Amsterdam, De Boelelaan 1108, 1081 HZ Amsterdam, the Netherlands

[c] KWR Water Research Institute, P.O. Box 1072, 3430 BB Nieuwegein, the Netherlands

Summary: 27 pages, 4 figures, 15 tables

Table of contents

	Page
**1. Sediment sampling and characterization**	
Text S1: Sediment sampling	S4
Figure S1: Overview of the two sites where the sediment for this study was collected	S4
Text S2: Sediment characterization	S4
Table S1: Characteristics and properties of the sediments	S5
Table S2: Grain size distribution of the sediments	S5
**2. Experimental setup**	
Text S3: DSW preparation	S6
Text S4: Artificial sediment preparation	S6
Figure S2: Schematical representation and photo of the experimental set-up	S6
**3. PFAS extraction and quantification in sediment, water and biota**	
Text S5: PFAS extraction and quantification protocol	S7
Text S6: Quality assurance and quality control	S8
Table S3: List of native PFAS analysed and their corresponding mass-labelled standards	S10
Table S4: Sample recoveries	S12
Table S5: Sample matrix effects	S12
Table S6: Limits of Quantification (LOQs)	S13
**4. PFAS concentrations**	
Table S7: PFAS concentrations for all matrices at the start of the mesocosm experiment	S14
Table S8: PFAS concentrations for all matrices in the absence of worms at the end of the mesocosm experiment	S15
Table S9: PFAS concentrations for all matrices in the presence of worms at the end of the mesocosm experiment	S17
**5. Mass Balance**	
Text S7: Calculations and uncertainties around our mass balance analysis	S19
Table S10: Average mass recovery for the contaminated set-up in the absence and presence of worms	S19
**6. PFAS Bioaccumulation factors**	
Table S11: Biota to sediment bioaccumulation factors (BSAFs) for the uptake of PFAS by the roots	S20
Table S12: Biota to sediment bioaccumulation factors (BCFs) for the uptake of PFAS by the shoots	S21
Table S13: Biota to sediment bioaccumulation factors (BSAFs) for the uptake of PFAS by the worms	S22
Table S14: Biota to sediment bioaccumulation factors (BSAFs) for roots and worms and bioconcentration factors (BCFs) for shoots, using only the final (t=56 d) PFAS concentrations in sediment and water	S23
Table S15: Comparison Biota to sediment bioaccumulation factors (BSAFs) for worms with those reported in previous studies	S24
**7. Bioaccumulation in plants versus animals**	

Figure S3: Bioaccumulation of PFAS from sediment in macrophytes versus worms	S25
**8. Bioturbation effect on the bioconcentration from water to shoots**	
Text S8: Bioturbation effect on the bioconcentration from water to shoots	S26
Figure S4: Effects of the presence of worms on the bioconcentration of PFAS in plant shoots	S26
**Reference list**	S27

1. Sediment sampling and characterization

Text S1: Sediment sampling

Gaasperplas in Amsterdam (The Netherlands; 52.308834 N, 5.001623 E) served as the reference site, where only diffuse pollution was expected since no known PFAS point source was present in proximity. The Blokkersdijk pond in Antwerp (Belgium; 51.231806 N, 4.342971 E), located approximately 200 m from the chemical company 3M Belgium, was selected as the contaminated site. All materials and equipment used for sampling were washed with water and cleaned with methanol. In the shallow zones of the lakes (depth < 1 m) sediments were sampled on 15th February 2022 and 21st February 2022 from the reference and contaminated site, respectively. In each lake, sediment cores (6 cm Ø; ~30 cm height) were collected within 3 meters from one another. After transport to the laboratory, the upper 10 cm from twelve intact cores per lake were transferred into short cores (6 cm Ø; ~14 cm height) to preserve the natural layering of the sediments. The cores were stored in a refrigerator at 4 °C for ~10 days before the start of the experiment, covered with parafilm to prevent drying of the sediment.

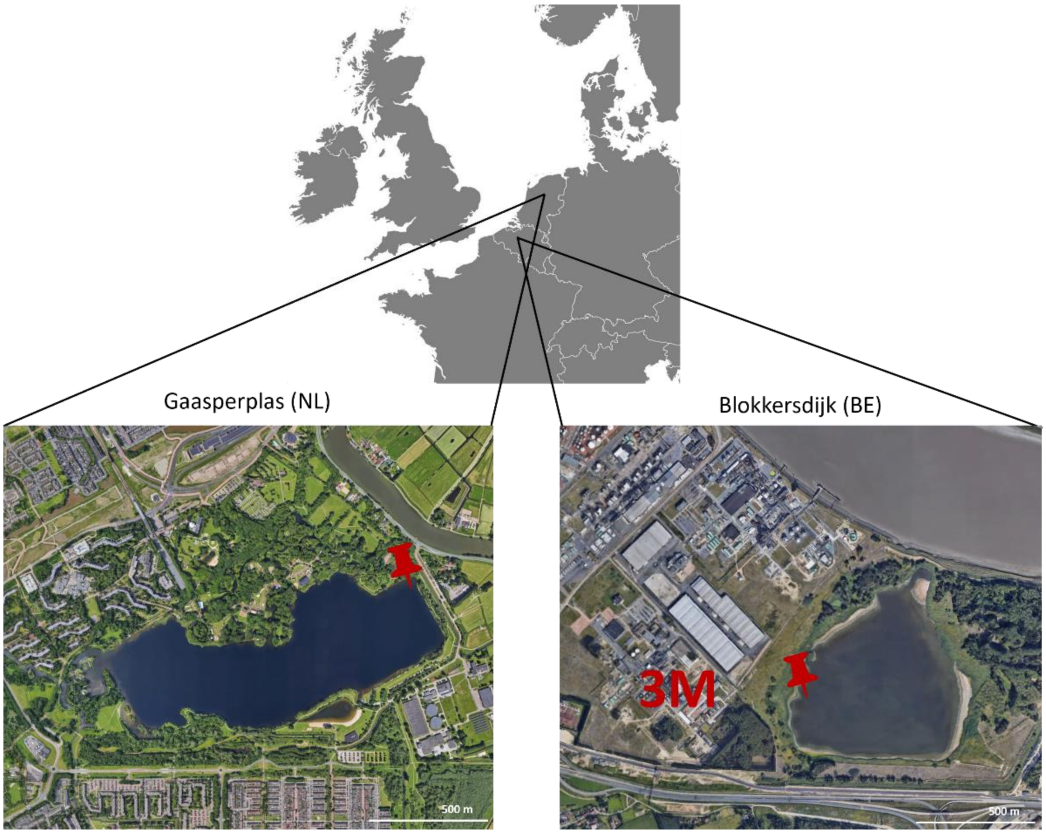

***Figure S1**: Overview of the two sites where the sediment for this study was collected, with the exact sampling points indicated by the red pins. Gaasperplas is the reference site, Blokkersdijk the PFAS-contaminated site. Images obtained from www.google.com/maps*

Text S2: Sediment characterization

For the sediment characterization, from each of the four cores per lake, a sample representative of the whole depth (10 mL) was collected by inserting a cut-off 25 mL serological pipet. Each sediment sample (n=4 per lake) was mixed, and a part was directly frozen for PFAS extraction to assess initial PFAS concentrations. Sediment samples were then freeze-dried, and grain size distribution was determined by separating a subsample of the sediment in seven size fractions (<63 to 2000 µm), after gently breaking the formed conglomerates using a metal spatula. The remaining sediment was milled to homogenize before further analyses.

Prior to organic Carbon (C) and Nitrogen (N) analysis, the sediments were acidified with sufficient HCl (3 mM) to remove the inorganic carbon. An elemental analyzer (Vario Isotope Cube, Elementar Analysensysteme GmbH, Langenselbold, Germany) was used to determine the percentage of C and N in the sediment samples, by correcting for the signal of the standards (sulfanilic acid) and blanks.

To determine the inorganic and total phosphorus content of the sediment, we used milled and redried (70 °C; 48 h) sediment directly and after heated it up (550 °C; 4 h), according to the protocol described by Blakemore et al. (1987). Briefly, 0.8 g dried sediment samples were extracted in 40 mL 0.5 M H_2SO_4 by shaking end-over-end for 16 h. Then, samples were centrifuged at 2000 rpm for 10 min and the supernatant was filtered through a 0.2 µm pore filter. A volume of 2 mL filtrate was transferred into new tubes, to which 1.6 mL of mixing solution, (5% Antimony Potassium Tartrate, 30% ascorbic acid, 25% ammonium heptamolybdate and 40% sulfuric acid) and 6.4 mL highly purified water (Milli-Q®) were added and left to react for 30 min. Absorption was measured using the Prove 300 Spectroquant® spectrophotometer and the concentration of phosphorus present in the sediment samples was calculated using calibration series with known concentrations of P_2O_5. By subtracting the results of the burned and unburned sediment samples, the concentration of organically bound phosphorus was determined.

Table S1: Characteristics and properties of reference (Gaasperplas) and PFAS-contaminated (Blokkersdijk) sediments, including organic carbon content (%), elemental nitrogen (%), total and organically bound phosphorus and mass-based C:N:P ratio. Results are reported as average and standard error of the mean (SEM) in between brackets (n=4).

	**Gaasperplas**	**Blokkersdijk**
**Organic C (%)**	0.19 (0.08)	0.29 (0.06)
**N (%)**	0.07 (0.01)	0.06 (0.01)
**Total P (mg/kg dw)**	7.6 (1.3)	34.4 (3.71)
**Organically bound P (mg/kg dw)**	1.48 (0.48)	9.39 (2.80)
**C: N: P ratio**	657: 215: 1	206: 41: 1

Table S2: Grain size distribution of reference (Gaasperplas) and PFAS-contaminated (Blokkersdijk) sediments. Results are expressed as % of the total dry sediment weight. The analysis was done using 4 field replicates (n=4).

**Size fraction (µm)**	**Gaasperplas**	**Blokkersdijk**
>2000	1.8 %	0.14 %
1000-2000	1.3 %	0.17 %
500-1000	5.5 %	0.30 %
250-500	40.4 %	2.4 %
125-250	46.4 %	63.7 %
63-125	3.8 %	31.9 %
<63	0.73 %	1.4 %

2. Experimental setup

Text S3: Artificial sediment preparation

The artificial sediment in which *Myriophyllum spicatum* was cultured, was prepared according to OECD test guideline No. 239 (OECD, 2014), with modifications and consisted of the following:

- 4% (dry weight) α-cellulose as organic matrix
- 20% (dry weight) kaolin clay (kaolinite content >30%)
- 76% (dry weight) quartz sand (>50% of the particles between 50 and 200 µm)
- Calcium carbonate ($CaCO_3$, 0.1 % dry weight) to buffer the pH of the final mixture of the sediment to 7.0 ± 0.5

Text S4: Dutch Standard Water (DSW) preparation

Each litre of DSW contained 2 mL of the following reagents:

- $CaCl_2 \bullet 2\ H_2O$ (100 g/L)
- $MgSO_4 \bullet 7\ H_2O$ (90 g/L)
- $NaHCO_3$ (50 g/L) + $KHCO_3$ (10 g/L)

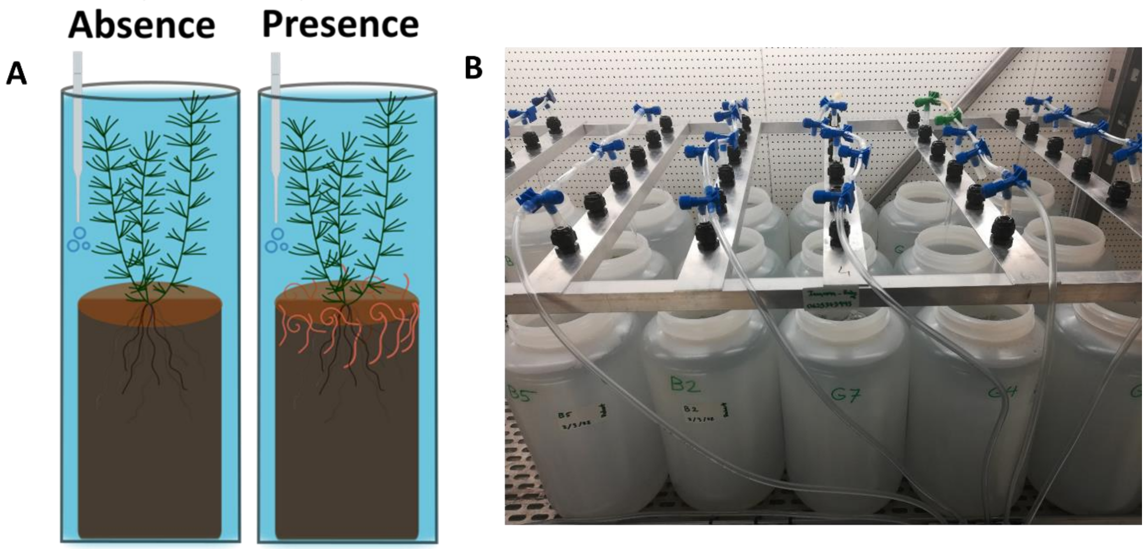

Figure S2*: Schematical representation (A) and photo (B) of the experimental set-up used to study the bioaccumulation of PFAS from reference and contaminated sediments by plants (*Myriophyllum spicatum*) and worms (*Lumbriculus variegatus*). Test cores containing* M. spicatum *shoots were immersed in 4 L HDPE bottles filled with DSW; worms were added to half the cores after 28 days of incubation. To maintain optimal conditions for the plants and the worms, a continuous aeration system was installed.*

3. PFAS extraction and quantification in sediment, water and biota

Text S5: PFAS extraction and quantification protocol

Native and isotopic mass labelled PFAS standards were purchased from Wellington Laboratories (Guelph, Canada), with the exception of n-deuteriomethylperfluoro-1-n-octanesulfonamidoacetic acid-d3 (N-MeFOSAA-d3, >99%) and n-ethylperfluoro-1-n-octanesulfonamidoacetic acid-d5 (NEtFOSAA-d5, >99%) that were purchased from Chiron (Trondheim, Norway), trifluoroacetic acid (TFA, >99%) and perfluoropropanoic acid (PFPrA, >97%) from Sigma-Aldrich (Zwijndrecht, Netherlands), perfluoroethane sulfonic acid (PFEtS, >98%) from Kanto Chemical (Japan), and n-methylperfluorobutanesulfonamide (>97%) from Apollo Scientific (Manchester, United Kingdom). ULC/MS grade methanol and LC-MS grade acetonitrile were acquired from Biosolve (Chimie, France). Ammonium acetate (>99%) and glacial acetic acid (>99%) were obtained from Sigma-Aldrich (Saint Louis, US), sodium hydroxide from Merck KGaA (Darmstadt, Germany) and ammonia solution (25%) from Thermo Fisher Scientific (Hampton, US). Milli-Q water was used in all experiments.

Water samples

All glassware used for the preparation and extraction of water samples was previously burned (20 min; 450 °C) to remove potential PFAS contamination. Prior to PFAS extraction, water samples were sonicated for 15 min and spiked with 10 µL of (0.1 – 0.2 ng/µL) mass-labelled extraction standard (ES). The pH was adjusted to 4, using acetic acid. PFAS were extracted from water samples using a weak anion exchange solid phase extraction (SPE) cartridge (Oasis® WAX, 3 cc, 60 mg, 60 µm; Waters Corporation Milford, USA). Before loading the samples, the SPE cartridges were preconditioned with 4 mL of 0.1% ammonium hydroxide in methanol, 4 mL methanol and 4 mL Milli-Q. After loading the samples, 4 mL of 25 mM acetate buffer solution (pH = 4) was used to wash the cartridges, which were then dried under vacuum for approximately 2 h. A polypropylene (PP) syringe filter (FilterBio®, 13 mm), pre-cleaned with methanol, was placed below each cartridge and PFAS were eluted using 4 mL of 0.1% ammonium hydroxide in methanol.

Sediment and biotic samples

Prior to PFAS extraction, the sediment and the biotic samples were freeze dried and subsequently milled with a mortar and pestle. Approximately 0.5 g and 0.1 - 0.3 g of dried, homogenized sediment and biota, respectively were transferred into a 50 mL PP centrifuge tube. PFAS were extracted using solid-liquid extraction, followed by solid phase extraction and an extra clean up step with activated carbon. The dried homogenized samples were then spiked with 10 µL of (0.1-0.2 ng/µL) mass-labeled extraction standard (ES). Solid-liquid extraction in combination with alkaline digestion was performed by adding 4 mL of acetonitrile and 2 mL of 2 mM sodium hydroxide in methanol to the samples. The samples were vortexed, sonicated for 15 minutes and centrifuged for 15 min at 4000 rpm. The supernatant was transferred into a clean 15 mL PP tube. These steps were repeated for 2 more rounds, but without sodium hydroxide, and with decreasing volumes of acetonitrile (3 mL and 2 mL, respectively). The supernatants were neutralized by dilution with Milli-Q to achieve a 70:30 ratio of aquatic to organic solvent. The pH of the samples was adjusted to 4 using acetic acid. For the solid phase extraction of sediment and biota samples, the same equipment and protocol as described for the water samples was followed. After the SPE, a microporous amorphous carbon molecular filter column (Supelclean ENVI-Carb™ 3 mL, 250 mg; Sigma Aldrich, Darmstadt, Germany) was used as an extra clean-up step to mitigate matrix effects. The activated carbon cartridges were first cleaned with 3 mL methanol. A PP syringe filter (FilterBio®, 13 mm), pre-cleaned with methanol, was added below each cartridge before loading the samples.

After extraction and clean-up, all samples (water, sediment and biota) were left to evaporate to 75 µL under nitrogen. Then, 175 µL of 0.05% acetic acid in Milli-Q and 10 µL of (0.1 ng/µL) mass-labeled injection standard solution (IS) were added. These extracts were vortexed and centrifuged for 5 min at 4000 rpm and transferred into a 250 µL vial. Samples were stored at -20 °C until further analysis.

PFAS quantification

PFAS were quantified with a Nexera UHPLC system (Shimadzu, Kyoto, Japan) coupled with a Bruker Daltonics MaXis 4G high resolution q-TOF-HRMS. Aliquots of 5 µL were injected on an Acquity UPLC CSH C18 column (130 Å, 2.1 x 150 mm, 1.7 µm). The flow rate was set at 0.2 mL/min and the column temperature was 50 °C. The mobile phase consisted of 0.05% acetic acid in Mili-Q (A) and 0.05 % acetic acid in acetonitrile (B). The eluent gradient started at 20% and was increased to 100% B using a linear ramp until 23 min, held for 3 min, and then reverted to initial conditions of 20% B. Internal mass calibration was performed with a 50 µM sodium acetate solution in a Mili-Q/methanol mixture (1:1, v/v), with a loop injection of 20 µL at the beginning of the analysis (0.1 − 0.5 min). Data obtained from the LC-MS/MS were processed with the TASQ software from Bruker Daltonics.

Text S6: Quality assurance and quality control

For each batch of samples, two procedural blanks and one quality control (QC) sample (spiked with native PFAS standards), were extracted simultaneously. The blanks and the QC of water samples contained Milli-Q, and for the sediment and biotic samples, pre-burned (450 °C; 20 min) inorganic sand (Silicon dioxide, by Sigma-Aldrich) was used. An isolator column (Waters Corporation Milford, USA) was installed after the solvent mixer of the LC pump and before the sample injector to separate any contamination originating from the LC system. Solvent blanks in the form of methanol injections were run between calibration curve points and samples to monitor potential carryover. Extraction recoveries were calculated for each sample using equation (1), in which $A_{ES\ sample}$ and $A_{ES\ standard}$ are the areas of the peaks of the extraction standard in the sample and standards, respectively, and $A_{IS\ sample}$ and $A_{IS\ standard}$ the areas of the peaks of the injection standard in the samples and standards, respectively. Table S2 lists the average recoveries of all mass-labelled analytes.

$$Recovery = \frac{A_{ES\ sample}}{A_{IS\ sample}} \div \frac{A_{ES\ standard}}{A_{IS\ standard}} \quad (1)$$

External calibration curves, consisting of concentration series ranging from 0.5 to 12,000 pg of native PFAS and a fixed amount of extraction and injection mass-labelled standards, were used for target analyte quantification, to evaluate the linearity (R^2 >0.99) of the calibration curves. In all cases, a minimum of 7 calibration data points were used for the quantification of the PFAS in the samples. Internal validation of the analytical method showed a <20 % relative standard deviation of the analysis results in relation to the quality control samples. Potential matrix effects (M.E.) on the ionization were evaluated by comparing the peak areas of injection standards in the samples and those in the calibration standards, prepared in solvent and Milli-Q, where no M.E. are expected. In the samples, no significant M.E. were observed (Table S3) and even in the cases where M.E was > 40 %, the sensitivity of the instrument was high and the peak area of the injection standard used was reliable (> 10^4) and could be used for quantification.

The limits of detection (LOD) and quantification (LOQ) were calculated separately for sediment, water and biota samples using equations (2) and (3), based on the signal of the analyte in the blanks (Barwick et al., 2014; Sadia et al., 2020).

$$LOD = [PFAS]_{Blanks} + 3 * StDev \quad (2)$$

$$LOQ = [PFAS]_{Blanks} + 10 * StDev \quad (3)$$

In these equations $[PFAS]_{blanks}$ corresponds to the average concentration of each analyte in the procedural blanks. For compounds that had no detectable signal in the blanks, the LOQ was assumed to be equal to the lowest data point of the calibration curve. If the calculated LOQ based on equation (3) was higher than the lowest calibration point, then that was considered as the LOQ. Compounds that were below the LOQ were excluded from further analysis and are counted as zero in the figures. Most compounds were not detected in the blank samples and therefore only the LOQs were reported (Table S4) as these had the same value as the LODs. Two compounds, PFBS and especially TFA, were present at considerable levels in the blanks, leading to elevated LOQs compared to the other compounds. Although high LOQs have previously been reported for TFA (Sadia et al., 2023), we decided to exclude both compounds from further analysis, to avoid bias in our conclusions. For the branched isomers of PFOA and PFHpS quantification was based on the calibration curve of their linear isomers (semi-quantification), due to the lack of analytical standards including the branched versions of these compounds at the time of analysis.

Table S3: List of native PFAS analysed in different matrices of the mesocosm study with reference (Gaasperplas) and contaminated (Blokkersdijk) sediments and their corresponding mass-labelled standards.

**Analyte**	**Acronym**	**Molecular Formula**	**CAS**	**Mass-labelled standard**
Perfluorocarboxylic acids				
Trifluoroacetic acid	TFA	$C_2HF_3O_2$	76-05-1	M4PFBA
Pentafluoropropionic acid	PFPrA	$C_3HF_5O_2$	422-64-0	M4PFBA
Perfluorobutyric acid	PFBA	C_4HF7O2	375-22-4	M4PFBA
Perfluoropentanoic acid	PFPeA	$C_5HF_9O_2$	2706-90-3	M5PFPeA
Perfluorohexanoic acid	PFHxA	$C_6HF_{11}O_2$	307-24-4	M5PFHxA
Perfluoroheptanoic acid	PFHpA	$C_7HF_{13}O_2$	375-85-9	M4PFHpA
Perfluorooctanoic acid – Linear	L-PFOA	$C_8HF_{15}O_2$	335-67-1	M8PFOA
Perfluorooctanoic acid – Branched	Br-PFOA			M8PFOA
Perfluorononanoic acid	PFNA	$C_9HF_{17}O_2$	375-95-1	M9PFNA
Perfluorodecanoic acid	PFDA	$C_{10}HF_{19}O2$	335-76-2	M6PFDA
Perfluoroundecanoic acid	PFUndA	$C_{11}HF_{21}O_2$	2058-94-8	M7PFUdA
Perfluorododecanoic acid	PFDoDA	$C_{12}HF_{23}O_2$	307-55-1	MPFDoA
Perfluorotridecanoic acid	PFTrDA	$C_{13}HF_{25}O_2$	72629-94-8	M7PFUdA
Perfluorotetradecanoic acid	PFTeDA	$C_{14}HF_{27}O_2$	376-06-7	M7PFUdA
Perfluorosulfonic acids				
Perfluoropropanesulfonic acid	PFPrS	$C_3HF_7O_3S$	423-41-6	M3PFBS
Potassium perfluoro-1-butanesulfonate	PFBS	$C_4HF_9O_3S$	375-73-5	M3PFBS
Sodium perfluoro-1-pentanesulfonate	PFPeS	$C_5HF_{11}O_3S$	2706-91-4	M3PFBS
Potassium perfluorohexanesulfonate – Linear	L-PFHxS	$C_6HF_{13}O_3S$	355-46-4	M3PFHxS
Potassium perfluorohexanesulfonate – Branched	Br-PFHxS			M3PFHxS
Sodium perfluoro-1-heptanesulfonate	L-PFHpS	$C_7HF_{15}O_3S$	375-92-8	M3PFHxS
Sodium perfluoro-1-heptanesulfonate – Branched	Br-PFHpS			
Potassium perfluorooctanesulfonate – Linear	L-PFOS	$C_8HF_{17}O_3S$	1763-23-1	M8PFOS
Potassium perfluorooctanesulfonate – Branched	Br-PFOS			M8PFOS
Potassium perfluoro-4-ethylcyclohexanesulfonate	PFECHS	$C_8HF_{15}O_3S$	646-83-3	M8PFOA
Sodium perfluoro-1-nonanesulfonate	PFNS	$C_9HF_{19}O_3S$	68259-12-1	M8PFOS
Sodium perfluoro-1-decanesulfonate	PFDS	$C_{10}HF_{21}O_3S$	335-77-3	M8PFOS
Sulfonamide-based precursors				
Perfluorobutylsulphonamide	FBSA	$C_4H_2F_9NO_2S$	30334-69-1	M3PFBS
Perfluorohexanesulfonamide	FHxSA	$C_6H_2F_{13}NO_2S$	41997-13-1	M3PFHxS
Perfluorooctanesulfonamide	FOSA	$C_8H_2F_{17}NO_2S$	754-91-6	M8PFOS
N-methylperfluorooctane sulfonamidoacetic acid – Linear	L-MeFOSAA	$C_{11}H_6F_{17}NO_4S$	2355-31-9	d5-N-EtFOSAA
N-methylperfluorooctane sulfonamidoacetic acid – Branched	Br-MeFOSAA	$C_{11}H_6F_{17}NO_4S$		d5-N-EtFOSAA
N-ethylperfluorooctane sulfonamidoacetic acid – Linear	L-EtFOSAA	$C_{12}H_8F_{17}NO_4S$	2991-50-6	d5-N-EtFOSAA
N-ethylperfluorooctane sulfonamidoacetic acid – Branched	Br-EtFOSAA	$C_{12}H_8F_{17}NO_4S$		d5-N-EtFOSAA
**Fluorotelomer-based precursors**				
Sodium 1H, 1 H,2H,2H-perfluoro-1-hexanesulfonate	4:2FTS	$C_6H_5F_9O_3S$	757124-72-4	M2-4:2FTS
Sodium 1 H, 1 H,2H,2H-perfluoro-1-octanesulfonate	6:2FTS	$C_8H_5F_{13}O_3S$	27619-97-2	M2-6:2FTS
Ether-containing compounds				
Sodium dodecafluoro-3H-4,8-dioxanonanoate	ADONA	$C_7H_2F_{12}O_4$	919005-14-4	M8PFOA
Potassium 9-chlorohexadecafluoro-3-oxanonane-1-sulfonate	9Cl-PF3OUDS	$C_8ClF_{16}KO_4S$	73606-19-6	M8PFOA
Potassium 11-chloroeicosafluoro-3-oxaundecane-1-sulfonate	11Cl-PF3OUDS	$C_{10}ClF_{20}HO_4S$	763051-92-9	M8PFOA
Perfluoro-4-oxapentanoic acid	PF4OPeA	$C_4HF_7O_3$	377-73-1	M4PFBA
Perfluro-5-oxahexanoic acid	PF5OHxA	$C_5HF_9O_3$	863090-89-5	M3PFHxA
Perfluoro-3,6-dioxaheptanoic acid	3,6-OPFHpA	$C_5HF_9O_4$	151772-58-6	M8PFOA
Potassium perfluoro(2-ethoxyethane)sulfonate	PFEESA	$C_4F_9HO_4S$	113507-82-7	M3PFHxS
Extraction standards				
Perfluoro-n-[^{13}C4]butanoic acid	M4PFBA	$^{13}C_4HF_7O_2$		
Perfluoro-n-[^{13}C5]pentanoic acid	M5PFPeA	$^{13}C_5HF_9O_2$		
Perfluoro-n-[1,2,3,4,6-^{13}C5]hexanoic acid	M5PFHxA	$^{13}C_5CHF_{11}O_2$		
Perfluoro-n-[1,2,3,4-^{13}C4]heptanoic acid	M4PFHpA	$^{13}C_4C_3HF_{13}O_2$		
Perfluoro-n-[^{13}C8]octanoic acid	M8PFOA	$^{13}C_8HF_{15}O_2$		
Perfluoro-n-[^{13}C9]nonanoic acid	M9PFNA	$^{13}C_9HF_{17}O_2$		
Perfluoro-n-[1,2,3,4,5,6-^{13}C6]decanoic acid	M6PFDA	$^{13}C_6C_4HF_{19}O_2$		
Perfluoro-n-[1,2,3,4,5,6,7-^{13}C7]undecanoic acid	M7PFUdA	$^{13}C_7C_4HF_{21}O_2$		
Perfluoro-n-[1,2-^{13}C]dodecanoic acid	MPFDoA	$^{13}C_2C_{10}HF_{23}O_2$		
Sodium perfluoro-1-[2,3,4-^{13}C3]butanesulfonate	M3PFBS	$^{13}C_3CHF_9O_3S$		
Sodium perfluoro-1-[1,2,3-^{13}C3]hexanesulfonate	M3PFHxS	$^{13}C_3C_3H2F_{13}O_3S$		
Sodium perfluoro-1-[^{13}C8]octanesulfonate	M8PFOS	$^{13}C_8HF_{17}O_3S$		
Sodium 1 H, 1 H,2H,2H-perfluoro-1-[1,2-^{13}C2]hexanesulfonate	M2-4:2FTS	$^{13}C_2C_4H_5F_9O_3S$		
Sodium 1 H, 1 H,2H,2H-perfluoro-1-[1,2-^{13}C2]octanesulfonate	M2-6:2FTS	$^{13}C_2C_6H_5F_{13}O_3S$		

Analyte	Acronym	Molecular Formula	CAS	Mass-labelled standard
N-Ethyl-n-perfluorooctanesulfonamidoacetic acId-d_5	d_5-N-EtFOSAA	$^2H_5C_{12}H_3F_{17}NO_4S$		
Injection standards				
Perfluoro-n-[2,3,4-^{13}C3]butanoic acid	M3PFBA	^{13}C 3CHF7O2		
Perfluoro-n-(1,2-^{13}C2]octanoic acid	M2PFOA	$^{13}C_2C_6HF_{15}O_2$		
Sodium perfluoro-1-[1,2,3,4-^{13}C4]octanesulfonate	M4PFOS	$^{13}C\ {}_4C_4HF_{17}O_3S$		
N-Methyl-n-perfiuorooctanesulfonamidoacetk acId-d_3	N-MeFOSAA-d_3	$^2H_3C_{11}H_3F_{17}NO_4S$		

Table S4: Average and standard error of the mean (SEM) sample recoveries of the mass-labelled standards in the different matrices of the mesocosm study with reference (Gaasperplas) and contaminated (Blokkersdijk) sediments.

Analyte	Sediment	Water	Biota
PFBA-M4	104% (1)	98% (1)	101% (1)
PFPeA-M5	65% (2)	98% (9)	61% (1)
PFHxA-M5	98% (6)	66% (4)	92% (4)
PFHpA-M4	120% (9)	83% (6)	94% (5)
PFOA-M8	95% (4)	86% (3)	90% (2)
PFNA-M9	92% (4)	104% (5)	90% (4)
PFDA-M6	106% (5)	99% (7)	105% (4)
PFUdA-M7	166% (7)	115% (17)	150% (11)
PFDoA-M	226% (43)	111% (34)	165% (43)
PFBS-M3	113% (7)	52% (6)	89% (6)
PFHxS-M3	114% (8)	82% (6)	85% (7)
PFOS-M8	89% (7)	63% (4)	70% (4)
4:2FTS-M2	108% (10)	75% (6)	114% (8)
6:2FTS-M2	83% (7)	83% (4)	87% (7)
EtFOSAA-d5	101% (12)	86% (11)	92% (3)

Table S5: Average and standard error of the mean (SEM) matrix effects of the mass-labelled standards in the different matrices of the mesocosm study with reference (Gaasperplas) and contaminated (Blokkersdijk) sediments

Analyte	Sediment	Water	Biota
PFBA-M3	17% (10)	45% (14)	-8% (7)
PFOA-M2	24% (6)	40% (14)	53% (5)
PFOS-M4	33% (6)	33% (11)	48% (4)
MeFOSAA-d3	-18% (12)	-34% (35)	-39% (23)

Table S6: Limits of Quantification (LOQs) for all native PFAS in different matrices of the mesocosm study with reference (Gaasperplas) and contaminated (Blokkersdijk) sediments

Analyte	Sediment (ng/g)	Water (ng/L)	Biota (ng/g)
11Cl-PF3OUDS	0.14	1.67	6.36
3-6-OPFHpA	0.02	0.22	0.84
4:2FTS	0.02	0.21	0.79
6:2FTS	0.02	0.21	0.80
9Cl-PF3ONS	0.02	0.21	0.79
ADONA	0.02	0.21	0.80
Br-EtFOSAA	0.004	0.05	0.19
Br-MeFOSAA	0.02	0.27	1.01
Br-PFHpS	0.02	0.21	0.80
Br-PFHxS	0.02	0.19	0.73
Br-PFOA	0.09	1.11	4.22
Br-PFOS	0.05	0.60	2.30
FBSA	0.09	1.11	4.22
FHxSA	0.02	0.22	0.84
FOSA	0.09	1.11	4.22
L-EtFOSAA	0.07	0.86	3.27
L-MeFOSAA	0.01	0.17	0.64
L-PFHxS	0.05	0.66	2.50
L-PFOS	0.22	2.65	10.07
MeFBSA	0.06	0.76	2.91
PF4OPeA	0.02	0.22	0.84
PF5OHxA	0.02	0.22	0.84
PFBA	0.09	1.04	3.97
PFBS	1.16	13.91	52.93
PFDA	0.02	0.22	0.84
PFDS	0.09	1.07	4.07
PFDoA	0.02	0.22	0.84
PFECHS	0.02	0.22	0.84
PFEESA	0.08	0.99	3.75
PFHpA	0.09	1.11	4.22
PFHpS	0.09	1.06	4.02
PFHxA	0.02	0.27	1.01
PFNA	0.02	0.22	0.84
PFNS	0.14	1.71	6.49
PFOA	0.16	1.88	7.17
PFPeA	0.02	0.22	0.84
PFPeS	0.02	0.21	0.79
PFPrA	0.09	1.11	4.22
PFPrS	0.08	1.01	3.86
PFTeDA	0.09	1.11	4.22
PFTrDA	0.02	0.22	0.84
PFUdA	0.09	1.11	4.22
TFA	186	2235	8504

4. PFAS concentrations in sediment, water and biota

Table S7: Average and standard error of the mean (SEM) individual PFAS concentrations for all matrices at the start of the mesocosm study with reference (Gaasperplas) and contaminated (Blokkersdijk) sediments. The analysis was done using 4 field/biological replicates (n=4), unless stated otherwise.

Analyte	Sediment (ng/g dw)		Water (ng/L)	Whole Plant (ng/g dw)	Worm (ng/g dw)
	Gaasperplas	Blokkersdijk			
11Cl-PF3OUDS	0.327 (0)[1]	<LOQ	<LOQ	<LOQ	<LOQ
3-6-OPFHpA	<LOQ	<LOQ	<LOQ	<LOQ	<LOQ
4:2FTS	<LOQ	<LOQ	<LOQ	<LOQ	<LOQ
6:2FTS	0.0985 (0.033)	<LOQ	<LOQ	3.21 (0.725)	<LOQ
8:2FTS	<LOQ	<LOQ	<LOQ	<LOQ	<LOQ
9Cl-PF3ONS	0.119 (0)	<LOQ	<LOQ	<LOQ	<LOQ
ADONA	<LOQ	<LOQ	<LOQ	<LOQ	<LOQ
Br-EtFOSAA	<LOQ	0.201 (0.014)	<LOQ	<LOQ	<LOQ
Br-MeFOSAA	0.0277 (0.002)	0.148 (0.012)	<LOQ	0.718 (0.066)	2.23 (0.284)
Br-PFHpS	<LOQ	0.131 (0.010)	<LOQ	<LOQ	<LOQ
Br-PFHxS	<LOQ	0.177 (0.007)	<LOQ	<LOQ	<LOQ
Br-PFOA	0.0233 (0.002)	<LOQ	0.333 (0.012)	0.607 (0.059)	1.12 (0.368)
Br-PFOS	<LOQ	2.57 (0.435)	<LOQ	<LOQ	<LOQ
FBSA	<LOQ	2.70 (0.328)	<LOQ	<LOQ	<LOQ
FHxSA	<LOQ	0.351 (0.033)	<LOQ	<LOQ	<LOQ
FOSA	0.284 (0.125)	2.76 (0.499)	<LOQ	2.85 (0.233)	4.08 (0.668)
L-EtFOSAA	<LOQ	2.27 (0.137)	<LOQ	<LOQ	<LOQ
L-MeFOSAA	<LOQ	0.636 (0.053)	<LOQ	<LOQ	<LOQ
L-PFHxS	0.0809 (0)	2.34 (0.088)	<LOQ	<LOQ	<LOQ
L-PFOS	0.498 (0.077)	8.78 (0.756)	2.169 (0.380)	3.83 (0.596)	<LOQ
MeFBSA	<LOQ	<LOQ	1.247 (0)	<LOQ	<LOQ
MeFOSA	90.3 (70.3)	32.2 (1.350)	<LOQ	163 (33.8)	87.3 (18.5)
PF4OPeA	<LOQ	<LOQ	<LOQ	<LOQ	<LOQ
PF5OHxA	<LOQ	<LOQ	<LOQ	<LOQ	<LOQ
PFBA	0.129 (0.019)	3.12 (0.109)	0.885 (0.162)	1.88 (0.203)	2.36 (0.688)
PFBS	<LOQ	3.55 (0.121)	<LOQ	<LOQ	<LOQ
PFDA	0.0966 (0.018)	0.053 (0.002)	<LOQ	0.694 (0.054)	0.999 (0.172)
PFDS	0.172 (0)	0.439 (0.051)	<LOQ	<LOQ	<LOQ
PFDoA	<LOQ	<LOQ	<LOQ	<LOQ	<LOQ
PFECHS	0.104 (0)	0.134 (0.006)	<LOQ	<LOQ	<LOQ
PFEESA	<LOQ	<LOQ	<LOQ	<LOQ	<LOQ
PFHpA	<LOQ	0.210 (0.006)	<LOQ	<LOQ	<LOQ
PFHpS	<LOQ	9.23 (0.351)	<LOQ	<LOQ	<LOQ
PFHxA	<LOQ	0.487 (0.009)	<LOQ	<LOQ	<LOQ
PFNA	0.0515 (0.002)	0.126 (0.005)	<LOQ	<LOQ	<LOQ
PFNS	<LOQ	0.461 (0.064)	<LOQ	<LOQ	<LOQ
PFOA	0.258 (0.072)	<LOQ	<LOQ	<LOQ	<LOQ
PFPeA	0.0366 (0.004)	0.281 (0.008)	<LOQ	0.718 (0)	1.87 (0.482)
PFPeS	<LOQ	0.482 (0.007)	<LOQ	<LOQ	<LOQ
PFPrA	<LOQ	2.81 (0.030)	0.272 (0.030)	<LOQ	5.50 (1.28)
PFPrS	<LOQ	<LOQ	<LOQ	<LOQ	<LOQ
PFTeDA	0.863 (0.560)	<LOQ	<LOQ	<LOQ	<LOQ
PFTrDA	0.812 (0)	<LOQ	<LOQ	<LOQ	<LOQ
PFUdA	<LOQ	<LOQ	<LOQ	<LOQ	<LOQ
TFA	<LOQ	<LOQ	<LOQ	<LOQ	1,265 (259)

1) Quantified in one replicate

Table S8: Average and standard error of the mean (SEM) individual PFAS concentrations for all matrices in the replicates without worms at the end of the mesocosm study with reference (Gaasperplas) and contaminated (Blokkersdijk) sediments. The analysis was done using 4 field/biological replicates (n=4), unless stated otherwise.

Analyte	Sediment (ng/g dw)		Water (ng/L)		Root (ng/g dw)		Shoot (ng/g dw)	
	Gaasperplas	Blokkersdijk	Gaasperplas	Blokkersdijk	Gaasperplas	Blokkersdijk	Gaasperplas	Blokkersdijk
11Cl-PF3OUDS	<LOQ	<LOQ	<LOQ	<LOQ	<LOQ	<LOQ	<LOQ	<LOQ
3-6-OPFHpA	<LOQ	<LOQ	<LOQ	<LOQ	<LOQ	<LOQ	<LOQ	<LOQ
4:2FTS	<LOQ	<LOQ	<LOQ	<LOQ	<LOQ	<LOQ	<LOQ	<LOQ
6:2FTS	<LOQ	<LOQ	0.931 (0)	<LOQ	<LOQ	<LOQ	<LOQ	48.2 (6.93)
8:2FTS	<LOQ	<LOQ	<LOQ	<LOQ	<LOQ	<LOQ	<LOQ	<LOQ
9Cl-PF3ONS	<LOQ	<LOQ	<LOQ	<LOQ	<LOQ	<LOQ	<LOQ	<LOQ
ADONA	<LOQ	<LOQ	<LOQ	<LOQ	<LOQ	<LOQ	<LOQ	<LOQ
Br-EtFOSAA	<LOQ	0.0716 (0.006)	<LOQ	1.06 (0.445)	<LOQ	0.592 (0)	<LOQ	3.70 (0)
Br-MeFOSAA	<LOQ	0.0523 (0.006)	<LOQ	0.677 (0.228)	<LOQ	1.07 (0.307)	<LOQ	1.37 (0.746)
Br-PFHpS	<LOQ	0.0335 (0.002)	<LOQ	<LOQ	<LOQ	0.971 (0)	<LOQ	3.14 (0)
Br-PFHxS	<LOQ	0.0623 (0.007)	<LOQ	1.48 (0.128)	<LOQ	1.71 (0)	<LOQ	<LOQ
Br-PFOA	<LOQ	0.141 (0.013)	0.410 (0.011)	2.33 (0.339)	1.93 (0.162)	1.58 (0.540)	<LOQ	1.79 (0)
Br-PFOS	<LOQ	7.78 (0.614)	1.32 (0.074)	30.5 (7.16)	<LOQ	134 (68.7)	<LOQ	84.1 (27.4)
FBSA	0.324 (0)[1]	0.712 (0.142)	2.01 (0)	47.2 (3.50)	73.2 (25.4)	49.1 (21.0)	<LOQ	18.5 (9.72)
FHxSA	0.130 (0)	0.160 (0.023)	<LOQ	<LOQ	33.4 (12.0)	19.3 (8.38)	5.95 (0)	8.20 (0)
FOSA	0.608 (0.164)	2.88 (1.052)	<LOQ	2.22 (0.884)	400 (121)	376 (151)	211 (80.6)	177 (121)
L-EtFOSAA	<LOQ	0.921 (0.091)	<LOQ	4.06 (1.01)	<LOQ	16.1 (3.91)	<LOQ	19.3 (5.20)
L-MeFOSAA	<LOQ	0.341 (0.060)	<LOQ	2.54 (0.584)	<LOQ	3.15 (1.27)	<LOQ	10.6 (4.54)
L-PFHxS	<LOQ	0.631 (0.074)	<LOQ	22.8 (2.39)	<LOQ	12.6 (4.69)	<LOQ	15.9 (1.65)
L-PFOS	0.0871 (0.007)	8.27 (0.227)	3.89 (0.250)	108 (18.9)	12.3 (2.08)	353 (109)	14.1 (4.65)	396 (181)
MeFBSA	<LOQ	<LOQ	1.22 (0.120)	<LOQ	<LOQ	<LOQ	<LOQ	8.40 (1.95)
MeFOSA	<LOQ	<LOQ	<LOQ	<LOQ	<LOQ	<LOQ	<LOQ	9.79 (0)
PF4OPeA	<LOQ	<LOQ	<LOQ	<LOQ	<LOQ	<LOQ	<LOQ	<LOQ
PF5OHxA	<LOQ	<LOQ	<LOQ	<LOQ	<LOQ	<LOQ	<LOQ	<LOQ
PFBA	0.0480 (0.002)	0.764 (0.049)	2.65 (0.262)	89.2 (2.80)	18.1 (9.06)	6.14 (3.02)	7.861 (2.48)	9.19 (1.88)
PFBS	<LOQ	2.30 (0.055)	<LOQ	83.2 (2.45)	<LOQ	<LOQ	113 (0)	<LOQ
PFDA	0.0179 (0)	0.0201 (0.001)	0.655 (0.071)	<LOQ	1.53 (0.023)	0.792 (0)	2.42 (0.702)	3.00 (1.23)
PFDS	<LOQ	0.096 (0.021)	<LOQ	0.820 (0)	<LOQ	3.69 (1.20)	<LOQ	12.7 (4.44)
PFDoA	<LOQ	<LOQ	<LOQ	<LOQ	<LOQ	<LOQ	<LOQ	<LOQ
PFECHS	<LOQ	0.0556 (0.004)	<LOQ	1.45 (0.337)	<LOQ	1.53 (0.608)	<LOQ	<LOQ
PFEESA	<LOQ	<LOQ	<LOQ	<LOQ	<LOQ	<LOQ	<LOQ	<LOQ
PFHpA	<LOQ	<LOQ	<LOQ	5.01 (0.408)	<LOQ	<LOQ	<LOQ	<LOQ

Analyte	Sediment (ng/g dw)		Water (ng/L)		Root (ng/g dw)		Shoot (ng/g dw)	
	**Gaasperplas**	**Blokkersdijk**	**Gaasperplas**	**Blokkersdijk**	**Gaasperplas**	**Blokkersdijk**	**Gaasperplas**	**Blokkersdijk**
PFHpS	<LOQ	0.200 (0.031)	<LOQ	12.1 (3.99)	<LOQ	13.8 (0)	<LOQ	25.1 (0)
PFHxA	<LOQ	0.176 (0.011)	2.09 (0.123)	16.4 (0.668)	<LOQ	<LOQ	<LOQ	<LOQ
PFNA	<LOQ	0.0460 (0.001)	0.456 (0.000)	0.677 (0)	<LOQ	1.23 (0)	<LOQ	6.46 (0)
PFNS	<LOQ	<LOQ	<LOQ	0.726 (0.277)	<LOQ	<LOQ	<LOQ	6.77 (2.56)
PFOA	<LOQ	0.703 (0.063)	4.94 (2.45)	15.3 (2.47)	49.5 (7.78)	6.75 (2.45)	<LOQ	28.1 (3.00)
PFPeA	0.0196 (0)	0.0970 (0.006)	0.641 (0.033)	14.2 (0.631)	2.67 (0.722)	1.37 (0.703)	2.39 (1.08)	1.75 (0)
PFPeS	<LOQ	0.0613 (0.006)	<LOQ	5.48 (0.317)	<LOQ	1.06 (0)	<LOQ	<LOQ
PFPrA	0.0739 (0.002)	0.522 (0.027)	1.02 (0.071)	0.748 (0.045)	10.7 (3.22)	6.89 (2.42)	9.13 (3.31)	<LOQ
PFPrS	<LOQ	<LOQ	<LOQ	3.57 (0.423)	<LOQ	<LOQ	<LOQ	<LOQ
PFTeDA	<LOQ	<LOQ	<LOQ	3.83 (0)	<LOQ	<LOQ	<LOQ	<LOQ
PFTrDA	<LOQ	<LOQ	<LOQ	<LOQ	<LOQ	<LOQ	<LOQ	12.1 (0)
PFUdA	<LOQ	<LOQ	<LOQ	<LOQ	<LOQ	<LOQ	<LOQ	<LOQ
TFA	<LOQ	<LOQ	<LOQ	<LOQ	<LOQ	<LOQ	<LOQ	<LOQ

1) Quantified in one replicate

Table S9: Average and standard error of the mean (SEM) individual PFAS concentrations for all matrices in the replicates with worms at the end of the mesocosm study with reference (Gaasperplas) and contaminated (Blokkersdijk) sediments. The analysis was done using 4 field/biological replicates (n=4), unless stated otherwise.

Analyte	Sediment (ng/g dw)		Water (ng/L)		Root (ng/g dw)		Shoot (ng/g dw)		Worm (ng/g dw)	
	Gaasperplas	Blokkersdijk	Gaasperplas	Blokkersdijk	Gaasperplas	Blokkersdijk[1]	Gaasperplas	Blokkersdijk[1]	Gaasperplas	Blokkersdijk[3]
11Cl-PF3OUDS	<LOQ	<LOQ	<LOQ	<LOQ	<LOQ	<LOQ	<LOQ	<LOQ	<LOQ	<LOQ
3-6-OPFHpA	<LOQ	<LOQ	<LOQ	<LOQ	<LOQ	<LOQ	<LOQ	<LOQ	<LOQ	<LOQ
4:2FTS	<LOQ	<LOQ	<LOQ	<LOQ	<LOQ	<LOQ	<LOQ	<LOQ	<LOQ	<LOQ
6:2FTS	0.076 (0.016)	<LOQ	0.490 (0.013)	0.912 (0.022)	<LOQ	<LOQ	<LOQ	<LOQ	<LOQ	<LOQ
8:2FTS	<LOQ	<LOQ	<LOQ	<LOQ	<LOQ	<LOQ	<LOQ	<LOQ	<LOQ	<LOQ
9Cl-PF3ONS	<LOQ	<LOQ	<LOQ	<LOQ	<LOQ	<LOQ	<LOQ	<LOQ	<LOQ	<LOQ
ADONA	<LOQ	<LOQ	<LOQ	<LOQ	<LOQ	<LOQ	<LOQ	<LOQ	<LOQ	<LOQ
Br-EtFOSAA	<LOQ	0.0725	<LOQ	1.33 (0.226)	<LOQ	<LOQ	<LOQ	1.16 (0)	<LOQ	<LOQ
Br-MeFOSAA	<LOQ	0.0820	0.453 (0.021)	0.711 (0.116)	5.77 (2.11)	16.0 (4.64)	2.44 (0.498)	2.11 (0.645)	1.49 (0.010)	<LOQ
Br-PFHpS	<LOQ	0.0304	<LOQ	<LOQ	<LOQ	<LOQ	<LOQ	<LOQ	<LOQ	<LOQ
Br-PFHxS	<LOQ	0.0446	<LOQ	1.43 (0.044)	<LOQ	<LOQ	<LOQ	<LOQ	<LOQ	1.11
Br-PFOA	0.0171 (0)[2]	<LOQ	0.281 (0.007)	2.18 (0.135)	2.48 (0.822)	7.90 (2.22)	1.18 (0.189)	1.47 (0.503)	<LOQ	<LOQ
Br-PFOS	<LOQ	2.02 (0.155)	<LOQ	30.4 (3.604)	<LOQ	101 (8.993)	<LOQ	58.3 (34.6)	<LOQ	79.9
FBSA	0.160 (0.002)	0.300 (0.067)	<LOQ	49.4 (3.445)	15.1 (0)	36.1(11.7)	<LOQ	<LOQ	<LOQ	6.16
FHxSA	0.0982 (0)	0.123 (0.00)	<LOQ	<LOQ	<LOQ	15.8 (9.06)	<LOQ	<LOQ	<LOQ	<LOQ
FOSA	0.409 (0.082)	2.26 (0.344)	<LOQ	3.24 (0.701)	46.6 (22.4)	165 (19.7)	12.9 (0.685)	23.7 (8.29)	3.97 (0.442)	<LOQ
L-EtFOSAA	<LOQ	0.666 (0.098)	<LOQ	5.43 (0.344)	<LOQ	<LOQ	<LOQ	11.40 (0)	<LOQ	<LOQ
L-MeFOSAA	<LOQ	0.222 (0.054)	<LOQ	3.13 (0.185)	<LOQ	<LOQ	<LOQ	<LOQ	<LOQ	<LOQ
L-PFHxS	<LOQ	0.684 (0.071)	<LOQ	23.4 (1.16)	<LOQ	<LOQ	<LOQ	7.35 (0)	<LOQ	13.8
L-PFOS	0.106 (0.013)	7.99 (1.13)	1.50 (0)	108.0 (24.6)	<LOQ	417 (51.5)	3.82 (0)	150.2 (88.2)	34.0 (4.61)	552
MeFBSA	<LOQ	<LOQ	<LOQ	<LOQ	<LOQ	<LOQ	<LOQ	<LOQ	<LOQ	<LOQ
MeFOSA	<LOQ	18.1 (1.24)	13.9 (1.20)	<LOQ	102 (35.8)	<LOQ	2882 (2015)	286 (0)	112 (0)	<LOQ
PF4OPeA	<LOQ	<LOQ	<LOQ	<LOQ	<LOQ	<LOQ	<LOQ	<LOQ	<LOQ	<LOQ
PF5OHxA	<LOQ	<LOQ	<LOQ	<LOQ	<LOQ	<LOQ	<LOQ	<LOQ	<LOQ	<LOQ
PFBA	0.053 (0.005)	0.467 (0.028)	3.16 (0.704)	95.7 (6.54)	4.19 (1.48)	12.6 (3.01)	2.22 (0.321)	2.85 (0.966)	1.77 (0.157)	11.5
PFBS	<LOQ	1.70 (0.104)	<LOQ	94.7 (4.04)	<LOQ	<LOQ	<LOQ	<LOQ	<LOQ	<LOQ
PFDA	0.0201	0.0210	0.343 (0.035)	0.229 (0)	2.60 (0)	<LOQ	1.26 (0.138)	1.19 (0)	5.33 (0.375)	1.73
PFDS	<LOQ	0.0592	<LOQ	0.504 (0.083)	<LOQ	<LOQ	<LOQ	1.46 (0)	<LOQ	5.24
PFDoA	<LOQ	<LOQ	<LOQ	<LOQ	<LOQ	<LOQ	<LOQ	<LOQ	<LOQ	2.60
PFECHS	<LOQ	0.0509 (0)	<LOQ	1.48 (0.198)	<LOQ	<LOQ	<LOQ	<LOQ	<LOQ	<LOQ

Analyte	Sediment (ng/g dw)		Water (ng/L)		Root (ng/g dw)		Shoot (ng/g dw)		Worm (ng/g dw)	
	**Gaasperplas**	**Blokkersdijk**	**Gaasperplas**	**Blokkersdijk**	**Gaasperplas**	**Blokkersdijk[1]**	**Gaasperplas**	**Blokkersdijk[1]**	**Gaasperplas**	**Blokkersdijk[3]**
PFEESA	<LOQ	<LOQ	<LOQ	<LOQ	<LOQ	<LOQ	<LOQ	<LOQ	<LOQ	<LOQ
PFHpA	<LOQ	<LOQ	<LOQ	2.19 (0.084)	<LOQ	<LOQ	<LOQ	<LOQ	<LOQ	<LOQ
PFHpS	<LOQ	0.247 (0.045)	<LOQ	9.26 (1.804)	<LOQ	<LOQ	<LOQ	<LOQ	<LOQ	<LOQ
PFHxA	<LOQ	0.165 (0.013)	1.52 (0.155)	8.34 (0.899)	<LOQ	4.254 (0)	<LOQ	<LOQ	<LOQ	<LOQ
PFNA	<LOQ	0.0406 [illegible]	0.459 (0)	0.546 (0.000)	<LOQ	7.96 (2.24)	<LOQ	<LOQ	<LOQ	0.863
PFNS	<LOQ	<LOQ	<LOQ	0.623 (0.114)	<LOQ	<LOQ	<LOQ	<LOQ	<LOQ	<LOQ
PFOA	<LOQ	<LOQ	<LOQ	13.6 (1.054)	<LOQ	<LOQ	<LOQ	8.79 (0)	<LOQ	<LOQ
PFPeA	0.0198 (0)	0.0971 [illegible]	0.640 (0.040)	6.08 (0.605)	2.98 (0.905)	13.40 (3.94)	1.68 (0)	1.79 (0.487)	<LOQ	<LOQ
PFPeS	<LOQ	0.0460 (0)	<LOQ	5.40 (0.233)	<LOQ	<LOQ	<LOQ	<LOQ	<LOQ	<LOQ
PFPrA	0.0710 [illegible]	0.457 (0.034)	1.46 (0.068)	0.917 (0.083)	14.5 (3.51)	36.2 (8.81)	4.05 (0)	5.81 (1.63)	3.41 (0.244)	<LOQ
PFPrS	<LOQ	<LOQ	<LOQ	4.38 (0.262)	<LOQ	<LOQ	<LOQ	<LOQ	<LOQ	<LOQ
PFTeDA	<LOQ	0.108 (0)	<LOQ	3.42 (0.048)	<LOQ	<LOQ	<LOQ	<LOQ	20.2 (4.74)	9.91
PFTrDA	<LOQ	<LOQ	<LOQ	<LOQ	<LOQ	<LOQ	<LOQ	<LOQ	35.6 (8.79)	<LOQ
PFUdA	<LOQ	<LOQ	<LOQ	<LOQ	<LOQ	<LOQ	<LOQ	<LOQ	4.28 (0.011)	<LOQ
TFA	<LOQ	47.0 (1.377)	<LOQ	<LOQ	3,006 (869)	9,925 (3,047)	2,115 (623)	1,172 (369)	1,142 (223)	<LOQ

1) Data from three replicates

2) Quantified in one replicate

3) Data from one replicate

5. Mass balance

Text S7: Calculations and uncertainties around our mass balance analysis

Mass balance was estimated in the form of PFAS (g) recovery in the system at the end of the experimental period, compared to the mass at the start of the mesocosm experiment (equation 4). In the numerator, we have the product of the final PFAS concentrations times the total dry mass of the respective matrix (n=5) per compound, averaged from all replicates. In the denominator, we have the same calculation, but at the start of the experiment with the difference that now the matrices were four (n=4) instead of five. This is because at the start of the experiment, we only had macrophyte shoots, while the roots gradually developed during the experimental period and were only extracted after the 56 days of exposure/incubation. The denominator in the treatment with both organisms is based on only one replicate, since only one worm replicate from the contaminated sediment could be analysed. This prevented us from calculating the error propagation of the ratio.

To minimize uncertainty, mass balances were assessed only for compounds that could properly be quantified (>LOQ) in all matrices at the end of the experimental period (since at the start concentrations of most compounds were expected to be <LOQ). Since this requirement was fulfilled by very few compounds in the case of the reference sediment, we only present the results for the contaminated sediment (Table S10).

$$Recovery = \frac{\sum_{i=1}^{5}([PFAS]_{end} * Mass\ in\ setup)}{\sum_{i=1}^{4}([PFAS]_{start} * Mass\ in\ setup)} \quad (4)$$

We conclude that only for some compounds an acceptable PFAS mass recovery could be obtained. These deviations are to be expected, due to the following factors:

- Possible presence of multiple PFSA precursors (on top of the ones we quantified in this work), which lead to increased loads of PFSAs at the end of the experimental period.
- Loss of plant and worm biomass while harvesting, which increased the uncertainty and error of the calculations.
- Precursor (bio)transformation (externally/internally by organisms).
- (Bio)transformation of non-precursors PFAS (externally/internally by organisms).
- Forming of sea-spray-like aerosols due to aeration system

Table S10: Average mass recovery for the contaminated set-up in the absence and presence of worms

	Mass Recovery (absence)	Mass Recovery (presence)
Br-MeFOSAA	43%	
Br-PFHxS	45%	74%
Br-PFOS	320%	579%
FBSA		87%
FOSA	115%	134%
L-EtFOSAA	43%	
L-PFHxS	37%	71%
L-PFOS	107%	227%
PFBA	51%	87%
PFDA		75%
PFDS		42%
PFHxA	67%	92%
PFNA		66%
PFPeA	81%	93%
PFPeS	24%	38%

6. PFAS Bioaccumulation factors

Table S11: Average and standard error of the mean (SEM) biota to sediment bioaccumulation factors (BSAFs) [kg sediment dw / kg root dw] for the uptake of PFAS from the reference and contaminated sediment into the roots of *Myriophyllum spicatum* in the absence and presence of worms. The analysis was done using 4 field/biological replicates (n=4), unless stated otherwise**.** Blank cells indicate that no BSAFs could be calculated for these compounds.

Analyte	BSAF root (absence)		BSAF root (presence)	
	Gaasperplas	Blokkersdijk	Gaasperplas	Blokkersdijk[1]
Br-EtFOSAA		7.34 (0)[2]		
Br-MeFOSAA		18.3 (5.60)		206 (106)
Br-PFHpS		28.9 (0)		
Br-PFHxS		21.8 (0)		
Br-PFOA		10.7 (3.43)	143 (0)	
Br-PFOS		15.8 (7.56)		35.6 (10.87)
FBSA		70.6 (23.9)		57.3 (40.5)
FHxSA		109 (33.0)		233
FOSA	1,053 (453)	150 (64.5)	158 (80.5)	69.6 (25.0)
L-EtFOSAA		15.6 (3.69)		
L-MeFOSAA		10.7 (2.60)		
L-PFHxS		18.2 (5.88)		
L-PFOS	151 (32.1)	42.9 (13.3)		46.0 (13.7)
PFBA	360 (169)	7.55 (3.47)	80.6 (31.3)	18.8 (6.96)
PFDA	86.7 (0)	36.5 (0)	118 (0)	
PFDS		20.1 (5.11)		
PFECHS		25.4 (8.34)		
L-PFHpS		79.7 (0)		
PFHxA				12.1 (8.55)
PFNA		25.3 (0)		151 (61.9)
L-PFOA		9.65 (2.786)		
PFPeA	135 (34.7)	13.3 (6.47)	147 (42.8)	109 (48.8)
PFPeS		13.7 (0)		
PFPrA	143 (41.4)	12.7 (4.12)	199 (39.3)	59.2 (23.6)

1) Data from three replicates

2) Quantified in one replicate

Table S12: Average and standard error of the mean (SEM) bioconcentration factors (BCFs) [L water / kg shoot dw] for the uptake of PFAS from the Gaasperplas and Blokkersdijk sediment into the shoots of *Myriophyllum spicatum* in the absence and presence of worms (*Lumbriculus variegatus*). The analysis was done using 4 field/biological replicates (n=4), unless stated otherwise. Blank cells indicate that no BCFs could be calculated for these compounds. Values >5000 L/kg ("very bioaccumulative" criterion set by ECHA) are coloured in red.

**Analyte**	**BCF shoot (absence)**		**BCF shoot (presence)**	
	**Gaasperplas**	**Blokkersdijk**	**Gaasperplas**	**Blokkersdijk[1]**
Br-EtFOSAA		6,592 (0)		746 (0)
Br-MeFOSAA		3,263 (2,227)	5,330 (980)	3,568 (1,211)
Br-PFOA		976 (0)	4,258 (760.8)	653 (194)
Br-PFOS		3,646 (1,511)		1,700 (912)
FBSA		356 (192)		
FOSA		97,695 (39,815)		14,305 (7,798)
L-EtFOSAA		6,232 (1,922)		1,829 (0)
L-MeFOSAA		5,155 (3,000)		
L-PFHxS		679 (9.00)		272 (0)
L-PFOS	3,583 (1,047)	4,117 (1,469)	2,558 (0)	1,445 (644)
PFBA	3,726 (1,499)	105 (23.3)	805 (184)	32.0 (10.5)
PFDA	3,424 (635)		3,846 (653)	5,199 (0)
PFDS				2,829 (0)
L-PFHpS		3,641 (0)		
PFNS		18,231 (8,325)		
L-PFOA		2,340 (498.3)		564 (0)
PFPeA	4,684 (1,865)	107 (0)	2,759 (0)	354 (117)
PFPrA	10,844 (4,320)		2,450 (0)	7,038 (2,070)

1) Data from three replicates

2) Quantified in one replicate

Table S13: Average and standard error of the mean (SEM) biota to sediment bioaccumulation factors (BSAFs) [kg sediment dw / kg worm dw] for the uptake of PFAS from the reference and contaminated sediment into *Lumbriculus variegatus*. The analysis was done using 4 field/biological replicates (n=4), unless stated otherwise. Blank cells indicate that no BSAFs could be calculated for these compounds.

**Analyte**	**BSAF worm**	
	**Gaasperplas**	**Blokkersdijk**[1]
Br-PFHxS		22.7
Br-PFOS		40.5
FBSA		13.4
FOSA	12.6 (3.87)	
L-PFHxS		26.5
L-PFOS	326 (38.9)	73.0
PFBA	35.4 (5.29)	21.9
PFDA	293 (1.79)	90.9
PFDS		54.1
PFNA		21.8
PFPrA	47.99 (2.79)	

1) Data from one replicate

Table S14: Average and Standard Error of the Mean (SEM) biota to sediment bioaccumulation factors (BSAFs) for roots [kg sediment dw / kg root dw] and bioconcentration factors (BCF) for shoots [L / kg shoot dw] for the uptake of PFAS by *Myriophyllum spicatum* from sediments and the overlying water from the reference and contaminated location, in the presence and absence of worms (*Lumbriculus variegatus*), and BSAFs for the worms [kg sediment dw / kg worm dw] using the average of the initial (t=0 d) and final (t=56 d) PFAS concentrations in sediment and water. The analysis was done using 4 field/biological replicates (n=4), unless stated otherwise. Blank cells indicate that no factors could be calculated for these compounds.

Analyte	BSAF root (absence)		BSAF root (presence)		BCF shoot (absence)		BCF shoot (presence)		BSAF worm	
	Gaasperplas	Blokkersdijk	Gaasperplas	Blokkersdijk	Gaasperplas	Blokkersdijk[1]	Gaasperplas	Blokkersdijk[1]	Gaasperplas	Blokkersdijk[3]
Br-EtFOSAA		4.20 (0)				13,185 (0)1		1492 (0)		
Br-MeFOSAA		10.4 (3.03)	416 (153)	151 (48.8)		6,526 (4,455)	10,659 (1,961)	7,136 (2,421)	108 (0.696)	
Br-PFHpS		11.8 (0)								
Br-PFHxS		13.4 (0)								9.85
Br-PFOA	146 (0)[2]	21.4 (6.85)	191 (73.5)			1,652 (0)		1,137 (344.6)		
Br-PFOS		24.1 (11.8)		42.8 (3.47)		7,292 (3,021)	3,862 (649.8)	3,400 (1,824)		35.2
FBSA		28.1 (11.4)		23.9 (6.68)		7,128 (383)				3.89
FHxSA		71.4 (27.6)		69.0 (36.6)						
FOSA	1,323 (570)	126 (44.9)	158 (80.1)	71.2 (9.44)		195,389 (79,631)		28,609 (15,596)	12.7 (2.84)	
L-EtFOSAA		9.73 (2.34)				12,464 (3,845)		3,658 (0)		
L-MeFOSAA		6.69 (2.41)				10,310 (6,000)				
L-PFHpS		2.94 (0)				7,283 (0)				
L-PFHxS		8.30 (3.02)				1,358 (18.0)		543.8 (0)		9.66
L-PFOA	298 (0)	19.3 (5.57)				4,681 (997)		1,129 (0)		
L-PFOS	44.4 (9.67)	41.5 (12.8)		53.3 (5.41)	4,605 (1,408)	8,047 (3,234)	2,087 (0)	2,826 (1,271)	112 (13.8)	67.5
PFBA		3.12 (1.52)	46.3 (16.8)	6.94 (1.66)	6,383 (1,761)	207 (46.2)	1,166 (217)	63.43 (20.9)	19.7 (2.10)	6.30
PFDA	29.1 (1.32)	21.2 (0)	43.9 (0)					10,398 (0)	99.8 (3.89)	48.1
PFDS		15.1 (5.63)			6,848 (1,270)		7,692 (1,305)	5,658 (0)		19.5
PFECHS		15.7 (5.91)								
PFHxA				12.8 (0)						
PFNA		14.1 (0)		95.8 (27.3)						10.4
PFNS						36,462 (16,650)				20.4
PFPeA	99.3 (33.1)	7.12 (3.62)	105 (31.4)	71.4 (21.5)		214 (0)		708 (234)		
PFPeS		3.78 (0)			9,368 (3,731)		5,519 (0)			
PFPrA	309 (107)	4.11 (1.43)	398 (78.5)	22.1 (5.48)				10,571 (3,057)	96.0 (5.58)	
PFPrS					20,553 (5,881)		4,207 (0)			
PFTeDA									46.9 (11.0)	
PFTrDA									87.6 (21.7)	

1) Data from three replicates

2) Quantified in one replicate

3) Data from one replicate

Table S15: Comparison with biota to sediment bioaccumulation factors (BSAFs) for worms reported in previous studies. Literature data reported on a wet-weight basis were transformed to a dry weight basis, assuming a 9% dry weight content of the worms and any normalizations to organic carbon (OC) content of the sediments were reversed to have the same units as the values calculated in this study [kg sed dw/kg worm dw][1]. In case a range of OC was reported, it was assumed that the lower BSAFs were obtained in sediments with the higher OC levels.

Analyte	Reference (avg)	Contaminated (avg)	Yun et al., 2023	Lasier et al., 2011 (lower end)	Lasier et al., 2011 (higher end)	Higgins et al., 2007
**PFBA**	35.4	21.9	339			
**PFNA**		21.8	1,101	50	4,444	2,146
**PFDA**	293	90.9	1,712	67	5,278	1,705
**L-PFHxS**		26.5	1,666	239	17,917	
**L-PFOS**	326	73	2,617	122	9,444	2,626
**PFDS**		54.1				795

1) estimated from reported BSAF values in kg sediment dw/kg worm ww, assuming a dw content of 9%

2) Field collected sediments

7. Bioaccumulation in plants versus animals

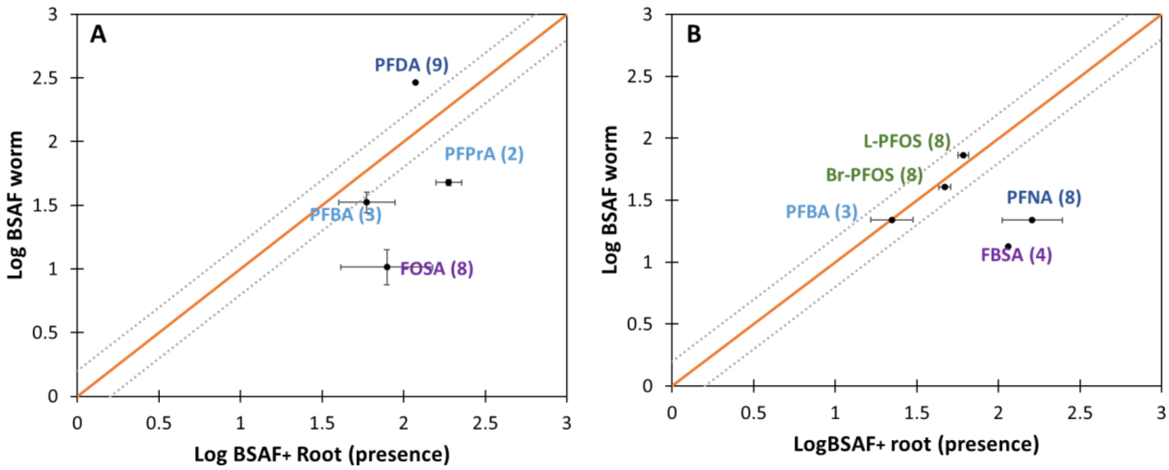

***Figure S3**: Bioaccumulation of PFAS from sediment in macrophytes (*Myriophyllum spicatum*) versus worms (*Lumbriculus variegatus*). Average (n = 4) log BSAFs for the plant roots in the presence of the worms are plotted against the log BSAFs for the worms after exposure to sediment from Gaasperplas (A) and Blokkersdijk (B). All BSAF values are expressed as kg sediment dw / kg root or shoot dw. The error bars represent the standard error of the mean. Missing error bars indicate that the compound was only quantified in one out of the four replicates. Names of the compounds corresponding to each data point are added and the number in parenthesis indicates the number of fluorinated carbons. The solid orange line indicates the 1:1 line (X = Y) and the dotted grey lines the margin of ± 0.2 log units, which was used to evaluate the distance of the compounds from the X = Y line.*

8. Bioturbation affects bioconcentration

Text S8: Effects of the presence of worms on the bioconcentration of PFAS from water to shoots

The shoot bioconcentration factors from water, expressed as BCF, in the absence and presence of worms, are compared in Figure S4. For the reference site, this comparison could be made for five compounds (Figure S4-A), with three of them positioned on or close to the 1:1 line and the other two positioned below it, indicating higher shoot uptake in the absence of the worm. For the contaminated sediment, the effect of the worm could be assessed for 11 compounds (Figure S4-B). There were three compounds, for which we could compare the BCF between the two treatments and the two locations. Two of them (L-PFOS and PFBA) exerted the same behaviour in both locations and were close to the 1:1 line, indicating no difference in the bioconcentration in the presence or absence of worms. For the remaining compounds, three out of the four precursors one long-chain PFCA (PFBA) and one long-chain PFSA (L-PFHxS), bioconcentrated stronger in the absence of worms and only PFPeA had higher BCF in the presence of worms. All other compounds were positioned on or relatively close to the 1:1 line, indicating no major effect due to the worm presence in the contaminated sediment.

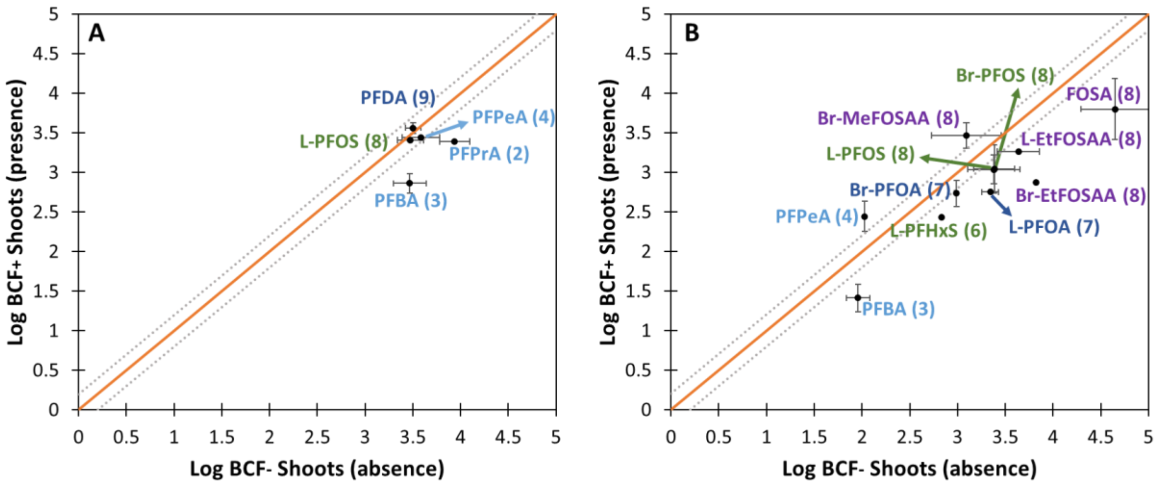

***Figure S4**: Effects of the presence of worms (*Lumbriculus variegatus*) on the bioconcentration of PFAS in plant shoots (*Myriophyllum spicatum*) from the overlying water of sediments from two field locations. Average Log BCFs for PFAS uptake in plant shoots in the presence and absence of the worms are plotted for Gaasperplas (A) and Blokkersdijk (B). BCFs are expressed as L / kg shoot dw. The error bars represent the standard error of the mean. Missing error bars indicate that the compound was only quantified in one out of the four replicates. Names of the compounds corresponding to each data point are presented and the number in parenthesis indicates the number of fluorinated carbons. The solid orange line indicates the 1:1 line and the dotted grey lines the margin of ± 20% error of the log transformed mean values, which was used to evaluate the distance of the compounds from the 1:1 line.*

Reference list

Blakemore, L. C., Searle, P. L., & Daly, B. K. (1987). Method for chemical analysis of soils. New Zealand Soil Bureau Scientific Report, 80. https://books.google.nl/books?id=pqDaRwAACAAJ

Higgins, C. P., Mcleod, P. B., Macmanus-Spencer, L. A., Luthy, R. G. (2007). Bioaccumulation of perfluorochemicals in sediments by the aquatic oligochaete *Lumbriculus variegatus*. *Environ. Sci. Tech*., *41* (13), 4600–4606; DOI 10.1021/es062792o

Lasier, P. J., Washington, J. W., Hassan, S. M., Jenkins, T. M. (2011).Perfluorinated chemicals in surface waters and sediments from northwest Georgia, USA, and their bioaccumulation in Lumbriculus variegatus. *Environ. Toxicol. Chem.*, *30* (10), 2194–2201; DOI 10.1002/etc.622

Organization for Economic Co-operation and Development (OECD) 2014. Test No. 239 Water-Sediment *Myriophyllum spicatum* Toxicity Test, OECD Guidelines for the Testing of Chemicals, Section 2, OECD Publishing, Paris, DOI 10.1787/9789264224155-en

Sadia, M., Nollen, I., Helmus, R., ter Laak, T. L., Béen, F., Praetorius, A., & van Wezel, A. P. (2023). Occurrence, Fate, and Related Health Risks of PFAS in Raw and Produced Drinking Water. *Environ. Sci. Tech*., 57(8), 3062–3074. DOI 10.1021/acs.est.2c06015

Yun, X., Lewis, A.J.; Stevens-King, G., Sales, C.M., Spooner, D.E., Kurz, M.J., Suri, R., McKenzie, E.R. (2023). Bioaccumulation of per- and polyfluoroalkyl substances by freshwater benthic macroinvertebrates: Impact of species and sediment organic carbon content. *Sci. Total Environ.*, *866*, 161208; DOI 10.1016/j.scitotenv.2022.161208.
